# Uncertainty in soil data can outweigh climate impact signals in global crop yield simulations

**DOI:** 10.1038/ncomms11872

**Published:** 2016-06-21

**Authors:** Christian Folberth, Rastislav Skalský, Elena Moltchanova, Juraj Balkovič, Ligia B. Azevedo, Michael Obersteiner, Marijn van der Velde

**Affiliations:** 1 Ecosystem Services and Management Program, International Institute for Applied Systems Analysis, 2361 Laxenburg, Austria; 2 Department of Geography, Ludwig Maximilian University, 80333 Munich, Germany; 3 Soil Science and Conservation Research Institute, National Agricultural and Food Centre, 82713 Bratislava, Slovak Republic; 4 School of Mathematics and Statistics, University of Canterbury, Christchurch 8140, New Zealand; 5 Department of Soil Science, Faculty of Natural Sciences, Comenius University, 84104 Bratislava, Slovak Republic; 6 European Commission, Joint Research Centre, 21027 Ispra, Italy

## Abstract

Global gridded crop models (GGCMs) are increasingly used for agro-environmental assessments and estimates of climate change impacts on food production. Recently, the influence of climate data and weather variability on GGCM outcomes has come under detailed scrutiny, unlike the influence of soil data. Here we compare yield variability caused by the soil type selected for GGCM simulations to weather-induced yield variability. Without fertilizer application, soil-type-related yield variability generally outweighs the simulated inter-annual variability in yield due to weather. Increasing applications of fertilizer and irrigation reduce this variability until it is practically negligible. Importantly, estimated climate change effects on yield can be either negative or positive depending on the chosen soil type. Soils thus have the capacity to either buffer or amplify these impacts. Our findings call for improvements in soil data available for crop modelling and more explicit accounting for soil variability in GGCM simulations.

## Abstract (web summary)

Global gridded crop models are increasingly used to assess climate change impacts on food production. Here, the authors assess crop yield uncertainty associated with soil data input, reporting that soil type strongly influences yield estimates, and may either buffer or amplify climate-related impacts.

## Graphical abstract

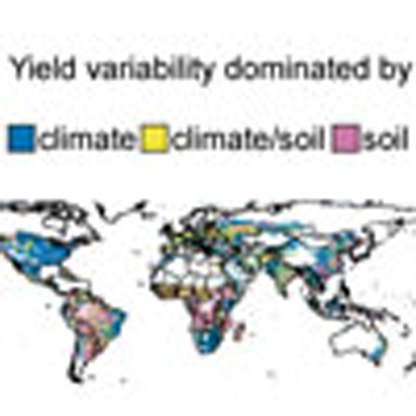

Crop growth simulation models have become an indispensable tool for estimating future impacts of climate change on crop yield^1^,^2^,^3^,^4^,^5^,^6^. Projections generally indicate adverse effects of climate change on crop yield at low latitudes and mixed effects at mid and high latitudes^2^,^6^. Uncertainties can be traced to different spatial and temporal sources including input data, model structure and process parameterization. The use of multiple general circulation model (GCM) projections has become the norm to characterize the uncertainty in climate projections for crop impact studies^1^,^2^,^3^,^4^,^5^,^6^. Recently, efforts have been made to characterize the uncertainty in crop model processes and setups themselves. Forcing an ensemble of seven global gridded crop models (GGCMs) with the same set of GCM projections resulted in comparable present-day yield levels but relative climate change impacts on crop yields ranged from about −40 to +25% by the 2090s for a high CO_2_ emission pathway^6^. Similarly, an ensemble of 27 field-scale wheat models exhibited a wide range of possible yield changes under high CO_2_ and temperature change of -100 to +100% in extreme cases^7^.

Surprisingly, soils have not been recognized as a key element in agricultural climate change impact studies on the global scale. The water and nutrient storage capacity of soils enables them to sustain crop growth during periods of adverse conditions and to either buffer^8^,^9^ or reinforce^10^ impacts of climate variability. Hence, investigating the uncertainty in global soil data and its impact on global crop simulations is essential. This uncertainty encompasses at least two major components, which are the quality of global soil data in terms of accuracy and range of measured soil characteristics on the one hand and the correct spatial allocation of soil types to cropland within GGCMs on the other.

Here we focus on the second component and its relevance for interactions between climate and soil characteristics in regulating plant growth functions. Soils are generally characterized by high spatial variability. Global soil data sets usually consist of maps delineating so-called soil mapping units (SMUs), representing regions of similar topography and soil genesis, and a database containing certain characteristics of a number of soil types. Thereby, one or several soil types can be linked to each SMU^11^,^12^. Alternatively, such databases provide data on selected soil characteristics that can be interpolated to a high spatial resolution^13^. As GGCMs usually run at a spatial resolution of 0.5° × 0.5° (about 50 × 50 km^2^ near the equator)^2^,^6^, various soil types or combinations of soil parameters may in both cases occur within one simulation unit. To our knowledge, GGCM studies generally use the soil that is dominant with respect to coverage^1^,^2^,^3^,^4^,^5^,^6^,^14^,^15^. In reality, it is not known *a priori* which one of these soil types is being used for agricultural production. Farmers are likely to grow their crops either on the soil most suitable for cultivation or choose the soil based on socio-economic considerations or limitations such as land tenure^16^.

A rigorous evaluation and GGCM sensitivity analysis to assess the importance of the uncertainty caused by soil data relative to climate input data and farm management is therefore crucial. The findings of recent regional studies that investigated how selection or extrapolation of soil data influences simulated crop yields are conflicting: Zhang *et al*.^17^ found that finer resolution soil data improve model performance in high-resolution crop simulations in the US mid-west, with only small differences in average crop yields but large deviations in the spatial representation of yields and carbon fluxes. By contrast, Angulo *et al*.^18^ found only marginal differences in simulated yields when aggregating soil data. They attributed their findings to high precipitation in the study region (Northwest Germany) and the algorithms used for estimating hydraulic parameters. Waha *et al*.^19^ finally concluded that the choice of crop model (APSIM or LPJmL) and climate data are greater sources of uncertainty than soil data in crop simulations for West Africa.

Here we used the Harmonized World Soil Database^12^ (HWSD; version 1.2 last updated 2012) to evaluate the impact of soil input data on yield estimates in a GGCM. Aggregating the HWSD to a 0.5° × 0.5° resolution resulted in unique combinations of up to 77 soil profiles per grid cell. The GGCM was based on the field-scale model EPIC^20^ (Environmental Policy Integrated Climate) and maize was selected as a representative crop. To account for effects of soil nutrient and water supply, we simulated a total of six management scenarios, consisting of no (no-nut), business as usual (bau-nut) or sufficient (high-nut) mineral fertilizer supply, each combined with purely rainfed cultivation or sufficient irrigation water supply. Yield variability was assessed using the coefficient of variation (CV, equation (7)). With respect to meteorological data, yields may vary between climate data sets in general and inter-annually due to inter-annual climate variability. Among different soil types, yields vary due to differences in nutrient availability, soil nutrient retention capacity and water-holding capacity. Examples of processes driven by both climate and soil factors are soil hydrology, soil temperature and evapotranspiration. To disentangle the signature of climate and soil on simulated yields, we compare the inter-annual yield variability solely associated with the dominant soil type (CV_dom_) to the variability resulting from the full range of soil types and annual yield estimates in each grid cell (CV_tot_). In this way, we can quantify the soil-related yield signals that are lost if only the dominant soil is used. The comparison of CV_dom_ and CV_soil_, which describes the variability of mean yields across all soil types in a grid cell, shows whether climate variability or uncertainty about the cultivation of soil types in the respective grid cells has a larger effect on yield estimates. The latter is primarily the case under agricultural low input conditions, whereas the first dominates under high input conditions and in regions with agriculturally adverse climates. We perform an assessment of the climate change impacts on simulated maize yield to highlight the importance of soil data for agricultural climate change impact studies. Soil data choices can cause differences in the magnitudes and - in extreme cases - also the direction of climate change impacts.

## Results

### Differences in climate- and soil-related yield variability

**Figure 1:**
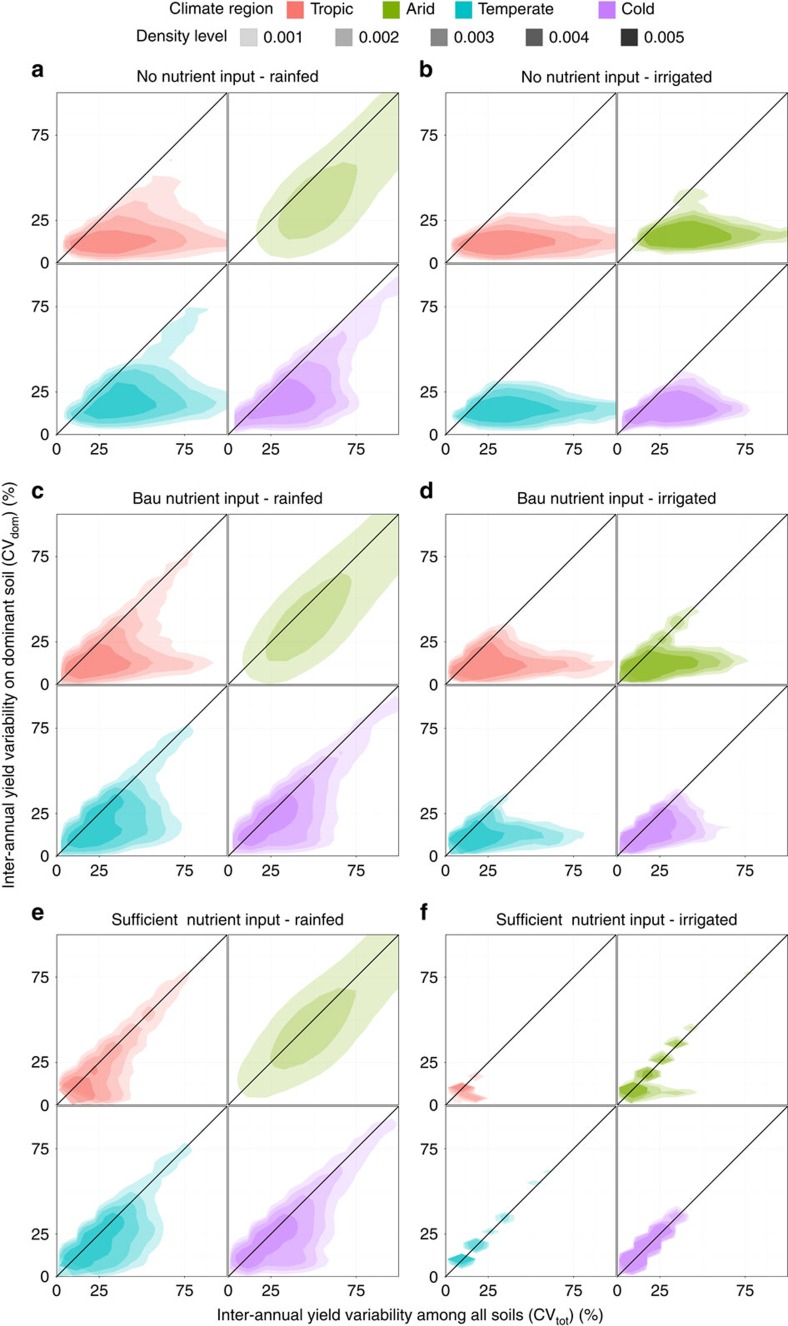
Coefficient of variation of maize yields in each grid cell for all soil types compared with the dominant soil only. Each panel depicts the coefficient of variation (CV) for a different nutrient and water management scenario: (**a**) no nutrient application and rainfed water supply, (**b**) no nutrient application and sufficient irrigation water supply, (**c**) business-as-usual (bau) nutrient application and rainfed water supply, (**d**) bau nutrient application and sufficient irrigation water supply, (**e**) sufficient nutrient application and rainfed water supply and (**f**) sufficient nutrient application and sufficient irrigation water supply (see Table 3 for details). The colours in the four subpanels displayed for each crop management scenario indicate the major Koeppen–Geiger climate regions^52^ (Supplementary Fig. 1 and Supplementary Table 1) tropic (red), arid (green), temperate (blue) and cold (purple). The size and shading of polygons of the same colour indicates levels of density corresponding to the bins shown in the density scale. The percentages of grid cells below the 1:1 line are shown in Table 1. Grid cells with only one reported soil type, arctic climate or without reported maize harvested area were excluded.

**Figure 2:**
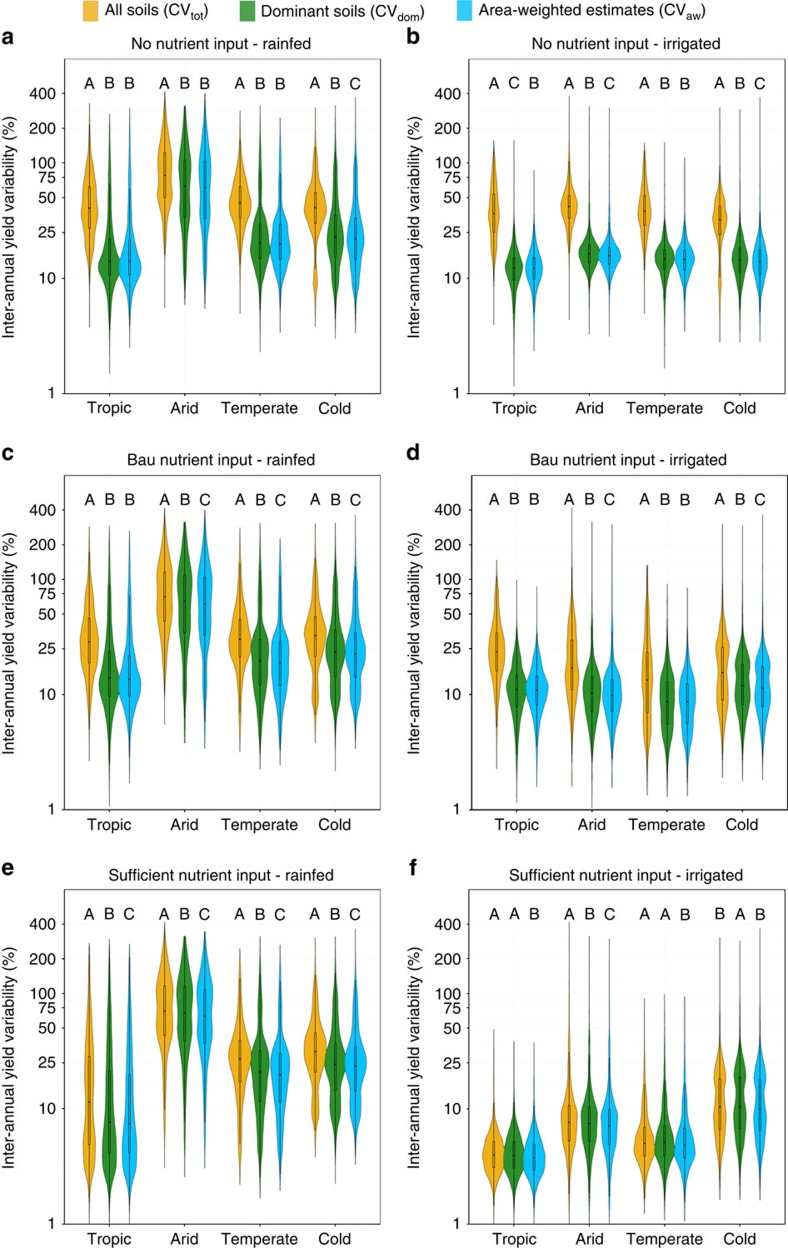
Violin plots of the coefficient of variation of maize yields for various soil pools and weightings. Coefficients of variation (CV) are shown for all soil types (CV_tot_), the dominant soil type only (CV_dom_) or the area-weighted CV across all soil types in a grid cell based on each soil's extent (CV_aw_). Each panel shows a nutrient and water management scenario with (**a**) no nutrient application and rainfed water supply, (**b**) no nutrient application and sufficient irrigation water supply, (**c**) business-as-usual (bau) nutrient application and rainfed water supply, (**d**) bau nutrient application and sufficient irrigation water supply, (**e**) sufficient nutrient application and rainfed water supply and (**f**) sufficient nutrient application and sufficient irrigation water supply (see Table 3 for details). The violins represent the density of values along the *y* axis and are of equal area. The capital letters A–C indicate whether the samples are significantly different according to a Tukey HSD test within each climate region and management scenario. The complete results of statistical analyses are presented in Supplementary Table 2. Grid cells with only one reported soil type, arctic climate or without reported maize harvested area were excluded.

The yield variability over a 10-year period and the total range of possible soil types CV_tot_ is substantially higher than the solely climate-driven 10-year yield variability CV_dom_ under most crop management configurations across the different climate regions (Fig. 1a–f and Table 1). This is particularly true under irrigated conditions without any exogenous nutrient application or with present-day fertilizer supply in low input regions (Fig. 1b,d). The impact of water deficit on yields is virtually eliminated under these conditions and soil nutrient supply becomes the main driver for yield variability. The soil ensemble-driven yield variability CV_tot_ and the climate-driven yield variability CV_dom_ are nearly identical if sufficient nutrients and irrigation water are supplied (Fig. 1f). By contrast, rainfed conditions raise both CV_dom_ and CV_tot_ substantially (Figs 1a,c,e and 2a,c,e) as precipitation patterns and soil hydrologic characteristics become major factors determining yield variability. This is most apparent in (semi-)arid regions with CVs often above 150%. In the absence of fertilizer supply, CV_tot_ is higher than CV_dom_ in more than 89% of all grid cells (Table 1). Medians of CV_tot_ and CV_dom_ (Supplementary Table 2) differ by factors of about 1.2–2.5 in arid and 1.8–3.1 in non-arid climates with larger differences in the rainfed than in the irrigated scenarios. Under such low-input conditions, crop nutrients are only supplied through the weathering of soil minerals and decomposition of soil organic matter (SOM)^21^ (atmospheric deposition is not taken into account due to lack of global data; see the Methods for details). The level of nutrient supply is least relevant in (semi-)arid regions where water supply and often adverse temperatures are the main factors limiting biomass production. The number of grid cells with CV_tot_>CV_dom_ decreases to ∼76% for the two bau-nut management scenarios (Table 1), representing present-day fertilizer application patterns with and without sufficient irrigation. This is accompanied by substantial decreases of CV_tot_ and to a lesser extent CV_dom_, especially under irrigated conditions (Figs 1c,d and 2c,d and Supplementary Table 2). Under rainfed conditions and with sufficient nutrient inputs (high-nut), CV_tot_ is larger than CV_dom_ in more than 61% of all grid cells. The application of sufficient irrigation water and nutrients reduces the differences between CV_tot_ and CV_dom_ to a minimum, rendering them statistically insignificant (Fig. 2f). In all cases, except where both nutrient and water supplies are sufficient, we find that CV_tot_ is significantly higher than CV_dom_ (Supplementary Table 2). We attribute this to the fact that inherent edaphic and climatic conditions that drive crop yields are less relevant when water and nutrients are supplied exogenously.

### Impact of area-weighting by soil extent on yield estimates

Recently, Zhang *et al*.^17^, in a regional high-resolution assessment of soil organic carbon sequestration, have tested the impact of weighting EPIC simulation results by soil type extent in each grid cell. The authors found that this weighting improved simulation results significantly. We have tested the impact of this approach as well by computing an area-weighted CV_aw_ (see the Methods for details). In our global study, the area weighted yield variability CV_aw_ or yield variability derived from the dominant soils alone (CV_dom_) often have very similar distributions (Fig. 2). They exhibit no statistically significant difference across various management scenarios and climate zones. The differences in the medians of CV_aw_ and CV_dom_ are smaller compared with the differences between one of the two and CV_tot_ (Supplementary Table 2). It needs to be stressed that area-weighting is a valuable approach if simulations refer to large coverage of grid cells as is the case in vegetation modelling or in high-resolution simulations of agricultural production in extensively cultivated regions. Globally, however, the area-weighted results do not provide a sufficient base for estimation of soil-induced yield variability in the GGCM approach. Even the soil type with the fifth largest extent has an area which is sufficiently large to account for all cultivated cropland area in >40% of all grid cells (Supplementary Fig. 2a). If only maize is taken into account, even the soil type with the 15th rank has sufficient coverage to account for the maize cultivated area in nearly 50% of all grid cells (Supplementary Fig. 2b). Therefore, it appears inevitable to take the whole range of soil types within in each grid cell into account, treating each one equally. Tentatively, this uncertainty may be bracketed by carrying out simulations for the dominant, most and least suitable soil types as was done here to highlight the importance of various subsets of soil types for model validation (see the Methods for details).

### Exogenous nutrient supply and soil-related yield variability

**Figure 3:**
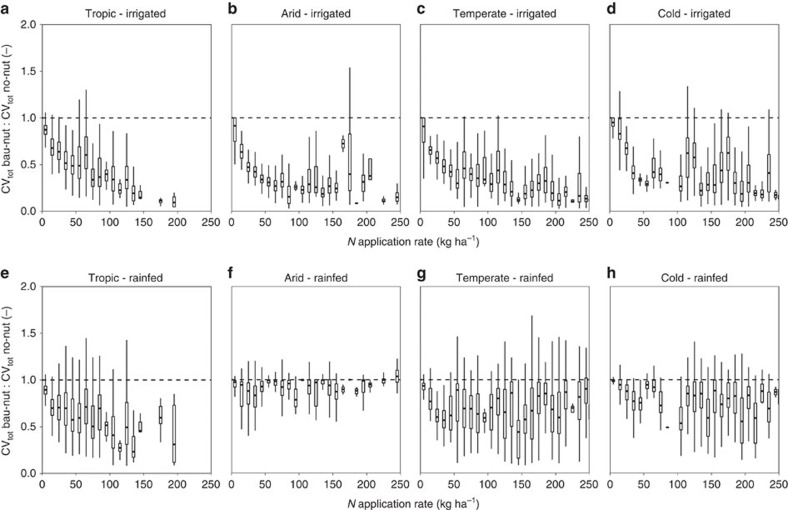
Boxplots showing the ratio of the coefficient of variation in the whole soil set for the business as usual compared with the no nutrient input scenario. Ratios of the coefficient of variation for the whole soil set (CV_tot_) for the business as usual (bau-nut) to no nutrient input (no-nut) scenarios are depicted for various Koeppen–Geiger climate regions and water management scenarios: (**a**) tropic and sufficient irrigation water supply, (**b**) arid and sufficient irrigation water supply, (**c**) temperate and sufficient irrigation water supply, (**d**) cold and sufficient irrigation water supply, (**e**) tropic and rainfed water supply, (**f**) arid and rainfed water supply, (**g**) temperate and rainfed water supply and (**h**) cold and rainfed water supply. The dashed line at intersect *y*=1 serves as a reference for CV_tot_ values in the no-nut scenario. Fertilizer application rates were binned in steps of 10 kg N ha^−1^. The extent of the *x* axis was limited to a maximum of 250 kg N ha^−1^ for better readability. Details on the fertilizer application rates are provided in the Methods section (Table 3) and Supplementary Fig. 3. Supplementary Fig. 1 shows the major Koeppen–Geiger climate regions as defined in Supplementary Table 1^52^. CV_tot_, the coefficient of variation for the whole soil set.

**Figure 4:**
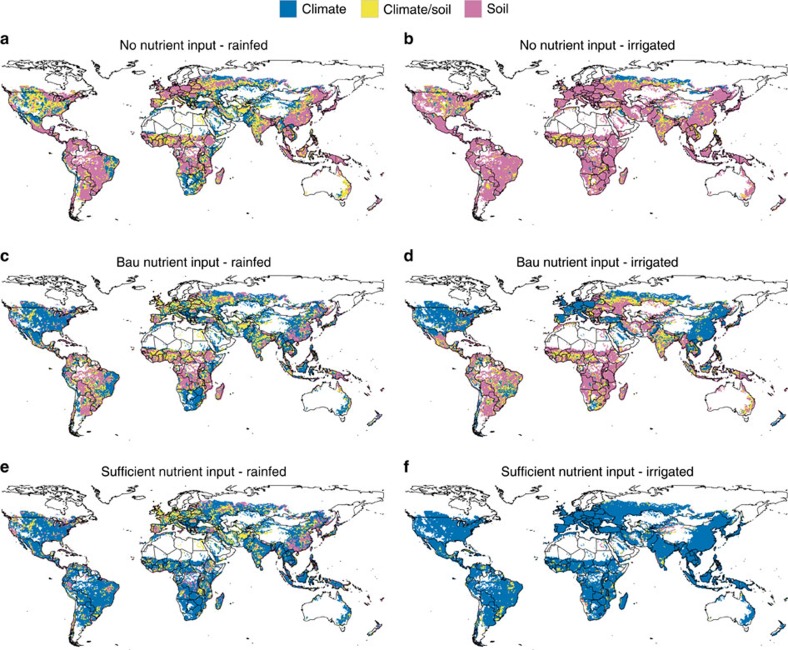
Grid cells in which climate or soil are dominating yield variability. Climate variability corresponds to CV_dom_ in Fig. 1 while yield variability due to choice of soil (CV_soil_) was computed from the 10-year means of yields from all respective soil types in each grid (see the Methods for details). Climate is considered to govern yield variability if CV_dom_/CV_soil_>30% (blue colour), soil is considered to govern yield variability if CV_soil_/CV_dom_>30% (magenta colour). Climate and soil are considered to govern yield variability jointly if they are in a range of ±30% of the respective larger value (yellow colour). Each panel represents a nutrient and water management scenario with (**a**) no nutrient application and rainfed water supply, (**b**) no nutrient application and sufficient irrigation water supply, (**c**) business-as-usual (bau) nutrient application and rainfed water supply, (**d**) bau nutrient application and sufficient irrigation water supply, (**e**) sufficient nutrient application and rainfed water supply, and (**f)** sufficient nutrient application and sufficient irrigation water supply (see Table 3 for details). Percentages of grid cells falling into either one of the categories are shown in Table 2. Grid cells with only one reported soil type, arctic climate or without reported maize harvested area were excluded.

As indicated above, the soil-induced yield variability depends strongly on nutrient management, especially if water is not a plant growth-limiting factor and temperatures are favourable for the cultivated crop. Under irrigated conditions, increasing fertilizer application rates amplify the difference in CV_tot_ among bau-nut and no-nut conditions (Fig. 3a–d). At high fertilizer application rates, this is particularly the case in tropical and temperate climates where temperatures are most favourable for maize cultivation and hence larger amounts of N can be utilized by the crop. By contrast, biomass production and therefore nutrient requirement is limited in arid and cold regions because of excessively high or low temperatures. Under rainfed conditions, such a trend is also observed albeit less pronounced than under irrigation (Fig. 3e–h). In temperate regions, the average ratio of CV_tot_ for bau-nut/no-nut increases from 0.35 when irrigated to 0.67 when rainfed (Supplementary Table 3). There are only minor differences between CV_tot_ in the no-nut and bau-nut scenarios in arid regions under rainfed conditions as yield variability is mainly climate driven (c.f. Fig. 4a,c). The mean ratio here is 0.91, but the two samples still differ significantly (Supplementary Table 3). These results emphasize the importance of soil type for crop yield estimates in GGCMs especially when it comes to assessments focusing on low input regions, which are considered the most vulnerable regarding present^16^ and potential future food security^6^.

### Spatial patterns in soil-related yield variability

Spatial patterns indicating whether soil or weather dominate yield variability depend strongly on crop management as well (Fig. 4). If irrigation water and fertilizer are not applied (Fig. 4a), then soils dominate yield variability in nearly 51% and weather in nearly 24% of all grid cells globally (Table 2). The latter is especially evident in (semi-)arid regions such as the Great Plains, Southern Africa, North-eastern Brazil and Central to West Asia. If irrigation water is supplied but fertilizer not (no-nut-irr), then soils dominate yield variability in 81% of the maize harvest area globally (Fig. 4b and Table 2). Yield variability in this case is driven by weather in only a few areas at the edge of agricultural suitability because of temperature limitations or if soil data indicates uniform soil characteristics. By contrast, soil-related yield variability is least important under high-nutrient input conditions. With solely rainfed water supply (Fig. 4e), differences in soil hydrological characteristics remain an important factor with yield variability dominated by soil type selection in 17.7% of all grid cells and no clear dominance of soil or climate in 17.9%. In irrigated agriculture (Fig. 4f), where also the remaining differences in soil hydrological characteristics are largely eliminated, soil type selection dominates yield variability in <2% of the grid cells (Table 2), which are located in the Brazilian savannah, southern Africa and around the Himalaya. This may appear to be in contrast to Fig. 4a,b, where under irrigation conditions soils are indicated to be important in more grid cells compared with rainfed conditions. However, it can be explained by endogenous nutrient supply from soils being a major plant growth-limiting factor besides climate under low-nutrient input conditions. As the model takes only the most limiting factor on each day of the growing season into account, nutrient stress may outweigh climate-related stresses under these circumstances (see the Methods for details). The rainfed and irrigated bau-nut managements (Fig. 4c,d) provide a mixed picture because of spatially explicit information on nutrient application. Most parts of the southern hemisphere (except Brazil, Argentina, Australia and New Zealand) resemble the pattern of the no-nut scenarios (Fig. 4a,b) as they presently lack substantial fertilizer inputs (Supplementary Fig. 3). The northern hemisphere shows the opposite, because of sufficient or even excessive fertilizer application rates in most parts such as in the United States, Europe and China^22^.

### The role of soils in climate change impact assessment

**Figure 5:**
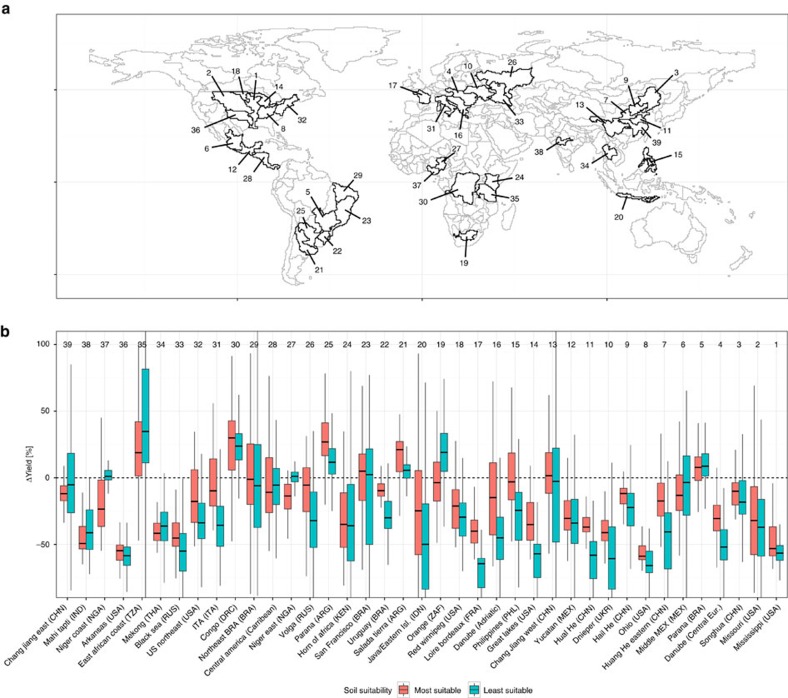
Projected percentage change in maize yields by the 2050s in the 39 largest food production units (FPUs). (**a**) FPUs as defined by intersecting water basins with major administrative boundaries^53^. (**b**) Relative change in maize yields for the presently most or least suitable soil type in each grid cell for the rainfed business-as-usual nutrient input scenario (bau-nut-rf; Table 3). The climate projection is based on HadGEM2-ES for RCP8.5 and is compared here with present-day climate data (see the Methods for details). Each of the selected FPUs comprises more than 10^6^ ha of harvested maize area. Ranking in terms of decreasing cultivated maize area is indicated by the order from right (largest) to left (smallest).

Projected changes in climate variables often exceed present weather variability in various regions globally and can even result in currently inexistent soil-climate combinations. This stresses the role of soils in attenuating or amplifying impacts of extreme weather or long-term climate shifts on crop yields. To highlight the importance of these climate–soil interactions, we performed an exemplary climate change impact assessment. The assessment focused on the world's major food production units (FPUs) using climate projections up to the 2050s performed with the HadGEM2-ES GCM under the RCP8.5 emission scenario (Fig. 5).

Depending on whether the highest or lowest yielding soil type is selected in each grid cell, estimated climate change impacts expressed relatively to the yield obtained under present climatic conditions can vary widely within a certain FPU. In 75% of the major FPUs, relative median yield losses are larger for the least suitable than for the most suitable soil types. This is partly due to the relative expression of the yield loss. For example, we find a nearly sixfold difference between the most (−5.5%) and the least (−32.1%) suitable soils of FPU 26 (Volga, RUS). The two subsets of soils provide yields of ∼2.9 and 1.4 t ha^−1^ during the baseline period, which results in absolute yield losses of 0.15 t ha^−1^ on the highest yielding soil types and 0.45 t ha^−1^ on the lowest yielding soil types. The difference in relative yield impacts among the least and most suitable soils is hence a product of the differences in absolute baseline yields (twofold) and absolute yield losses (threefold). In any case, as large-scale agricultural climate change impact assessments often focus on relative changes in agricultural productivity^1^,^2^,^3^,^6^, the specific selection of soil types for GGCM simulations can have a significant impact on the spatial interpretation of results. Besides the magnitude of the impact, even the direction (+/−) may be inversed as a function of soil type choice as is the case in FPUs 13, 19, 27 and 37.

## Discussion

Soil processes modulating impacts of climate on plant growth and crop yields are highly complex and to fully disentangle them is beyond the scope of this study. To shed light on some of these processes, we have explored plant growth dynamics and associated environmental variables in two contrasting grid cells (Supplementary Discussion 1 and Supplementary Figs 4–12). The evaluation reveals that dynamic interactions of soil texture, precipitation and plant water requirement can result in unexpected climate change impact responses. If precipitation decreases moderately, climate change can cause a more severe impact on crop yields in soils with lower water-holding capacity that are drained rapidly, whereas a soil of finer texture may buffer the potential deficit by storing water for longer periods. However, very low precipitation levels can also result in lower plant-available water in fine-textured soils. For such soils, only the very fine pore-space may be filled with water that is not readily accessible for plants or the fine texture can cause higher run-off^10^. This would result in a more vulnerable crop while a coarse textured soil can result in higher levels of plant available water. Besides these water-related processes, the nutrient supply of a soil can also attenuate or amplify climate change impacts by modulating the plant's sensitivity to climate variables (see the Methods for details) as has been discussed extensively above with respect to the nutrient management scenarios. Temperature, solar radiation and atmospheric CO_2_ concentration affect biomass accumulation independently from soils and soil processes in the model.

At the global scale, changes in maize yields vary from −26% on the most suitable soil to −27% on the dominant soil and −31% on the least suitable soil in the climate change scenario (not shown). This results in an absolute uncertainty range of 5%. A recent assessment based on yield estimates from 14 GGCMs forced with projections from 5 GCMs showed that the estimated change in maize yields may vary from −20 to +15% among crop models around the year 2050, whereas the GGCM used here (GEPIC) had an absolute uncertainty range of ∼15% when driven by the 5 GCMs^6^. This indicates that the largest source of uncertainties in agricultural climate change impact assessments lies in crop models themselves followed by uncertainties in GCM projections. Nevertheless, soil characteristics and data have an undeniable impact on uncertainty in estimates of crop yields under climate change. Thus, for the GGCM used here, the magnitude of the range in observed impacts due to the selection of soil data amounted to about a third of the uncertainty that was associated with the five GCM projections. Quantifying this relative importance in GGCM × GCM ensembles will require the direct inclusion of various soil data in future ensemble runs.

Further sources of uncertainty in global crop yield simulations are the correct spatial allocation of cropland, crop management and crop rotations besides varying algorithms applied by different crop growth simulation models. Several of these uncertainties are presently being addressed within the GGCM intercomparison project^23^. Porwollik *et al*. (in preparation), for example, found that depending on the cropland data set used for aggregation, global average maize yield estimates differ only slightly, but can range widely in single countries. A recent study based on the GEPIC model estimated that rotation of maize with a leguminous shrub and soil conservation practice can buffer adverse changes in precipitation under climate change as compared with conventional intensification^5^. Although such uncertainties are presently addressed individually, the contributions of single factors to overall model uncertainty and potential interactions will have to be investigated in multi-dimensional experiments.

Similar effects can be expected for other crop models with detailed representation of soil processes^6^,^24^, such as APSIM, DSSAT, DNDC or Century and for certain ecosystem and hydrologic models. The importance of soil data quality and allocation will increase in the future as the range of soil processes taken into account in field-scale models and GGCMs is constantly expanding^24^,^25^. Considering that yield estimates from bio-physical crop models often form the basis for subsequent agro-economic (climate change impact) assessments^26^, uncertainties and errors originating from soil data will propagate throughout the whole assessment chain from bio-physical to economic impacts and further to policy recommendations. This amplifies the need for developing new strategies on how to deal with soil-related uncertainty in GGCM simulations.

As any other soil mapping product, the HWSD itself has intrinsic uncertainties and quality limitations. For example, substantial differences in the characteristics and locations of soil types can be observed when comparing global databases like HWSD to finer-scale regional soil data^27^. Such soil mapping uncertainties can be further amplified when used in models as has been shown, for example, by Hendriks *et al*.^27^ and Lin *et al*.^28^ One way to avoid this issue is the use of soil profile information together with spatially represented covariates in digital soil mapping products to infer directly quantitative soil information at a given spatial resolution^13^.

Indeed, on-going efforts to improve the quality of basic soil data, for example, by increasing the coverage of soil samples and combining them with remote sensing (for example, http://globalsoilmap.net^29^) are essential. Yet, our results show that the correct spatial allocation of these soil data to present cropland is at least equally important. One project targeting the regional matching of soil data and cropland by merging ground and remote sensing data is presently carried out by the Africa Soil Information Service (http://africasoils.net). As long as there are no global high-resolution input data that match all required inputs (climate, soil, cropland and management) per grid, however, the full ensemble of contrasting soil types present in each location should be taken into account in GGCM simulations. Alternatively, the role of soils and their variability in buffering or amplifying future climate impacts on crops should at least be bracketed by simulating the most and least suitable soils under cultivation. Another key ingredient for improved climate impact assessments is the collection and GGCM representation of crop management practices. This and better characterizing soils and their variability in GGCMs will allow for identifying farm-level adaptation options tailored to the range of marginal to near-optimal conditions experienced in the field.

## Methods

### Model description

We used the geographic information system (GIS)-based global crop modelling framework GEPIC^30^ based on the field-scale model EPIC v. 0810 (refs 20, 25) for simulating crop production. Maize was used as a representative crop to model as it globally accounts for the largest production volume and the second largest harvested area. Concordantly, it is widely simulated with GGCMs^1^,^2^,^3^,^4^,^5^,^6^,^14^. GEPIC reads large-scale input data sets and runs EPIC for each grid cell of a defined area treating each grid cell as a field with specific climate, soil, topography and management. The spatial resolution applied here was 0.5° × 0.5°, which is presently the norm in global crop model simulations^6^. The last 10 simulation years after a spin-up period of 30 years were used for evaluation of the simulation results. EPIC uses the energy-to-biomass conversion approach for estimating potential biomass increase at a daily time step. The potential increase is subsequently adjusted by the major stress factor out of nutrients, water, temperature and aeration to an actual biomass gain. Besides crop growth, soil processes ranging from organic matter cycling to wind erosion are simulated. Soils in the EPIC model provide rooting space for plants and serve as the medium storing, cycling and providing nutrients and water. After crop uptake, drainage, leaching and other losses to the environment, both can be replenished by fertilizer and irrigation water application. To account for this, we simulated six management scenarios with varying levels of water and nutrient supply as shown in Table 3. The scenarios with ample irrigation and fertilization are assumed to eliminate water- and nutrient-related stresses on plant growth. The parameterization applied here has been specified in Folberth *et al*.^31^ and Rosenzweig *et al*.^6^ using mainly default model parameters. More detailed descriptions of model routines and further input data are provided at the end of the Methods section.

### Input data for climate and topography

Climate data from the WFDEI.GPCC database^32^ were provided through the ISI-MIP and AgGRID projects (http://isi-mip.org; http://agmip.org/ag-grid) at a resolution of 0.5° × 0.5°. WFDEI.GPCC is based on temperature and solar radiation from ERA-interim^33^ and precipitation and snowfall from GPCC^34^. Climate projections for HadGEM2-ES RCP8.5 were provided by ISI-MIP^35^ and are based on runs for the CMIP5 project^36^. Elevation and slope were derived from the digital elevation model GTOPO30 (ref. 37).

### Soil database processing and evaluation

The HWSD in its version 1.2 (ref. 12) was used for preparing soil input data for GEPIC. The HWSD is presently the most up-to-date global soil database, bringing together data from several national and regional soil assessments. Thereby, 45,000 SMUs were defined by local experts as homogenous soil regions to which certain soil types were attributed (Supplementary Fig. 13). Depending on the size of the SMU and the given soil heterogeneity, these can be up to ten different soil types with varying fractions of coverage. Exceptions are China and Greenland among other smaller units, for which only one soil type has been reported per SMU.

The mapping raster of the HWSD was aggregated from its native resolution of 30 arcsec to a 0.5° × 0.5° grid complying with the present state-of-the-art in global crop modelling. Thereby, all soil types from all SMUs in each grid cell were attributed to the respective grid cell. Their share was calculated based on the coverage of the SMU within the grid cell and the reported share of the soil type in the SMU (Supplementary Fig. 14). This resulted in up to 77 soil profiles in each grid (Supplementary Fig. 15), with the dominant soil type covering between <10% and 100% of each simulated grid cell (Supplementary Fig. 16). Owing to the small extent of SMUs in China, this approach resulted also here in various combinations of soils per grid cell because of intersecting SMUs (Supplementary Fig. 17 and Supplementary Fig. 15b).

The aggregation resulted also in smaller extents of the dominant soil type in each grid cell as shown in Supplementary Fig. 16. The dominant soil type in each grid cell would still be sufficient for covering all cropland in >80% of all grid cells (Supplementary Fig. 2a). For the subsequent soil types, this figure decreases continuously. However, even up to the fifth soil type more than 50% of all cropland could be allocated on the respective soil type in grid cells that have this number of soil types reported. This highlights that using the dominant soil type in each grid cell is not justified by the extent of cultivated areas in each grid cell. A similar and even more pronounced pattern can be observed if only the harvested area for maize is taken into account (Supplementary Fig. 2b).

### Crop nutrient management scenarios

Six nutrient and water management scenarios were simulated (Table 3). The business–as-usual (bau-nut) nutrient management scenario represents the norm in most GGCMs^6^ and is based on data representative for ‘around the year 2000'. Reported global planting dates and growing season lengths were obtained from the database by Sacks *et al*.^38^. Fertilizer application rates for N and P were provided by the GGCM intercomparison coordination team^23^. They are based on global, spatially explicit and crop-specific fertilizer application rates by Mueller *et al*.^39^ combined with nutrients embedded in manure. The application rates for N and P in this scenario are displayed in Supplementary Fig. 3 and range from 1 (parts of sub-Saharan Africa) to 372 (Egypt) kg N ha^−1^ a^−1^ and 0 (parts of sub-Saharan Africa) to 150 (New Zealand)  kg P ha^−1^ a^−1^. Although this management scenario is important as a reference for comparisons with other studies, the global imbalance in agricultural nutrient application rates limits the spatial comparability of soil- and climate-related yield variability.

Hence, two additional nutrient management scenarios were run for evaluating the effect of soil nutrient contents on crop yields (no-nut) or the effect of soil texture and water supply (high-nut). No nutrients were supplied in the no-nut scenario in order to capture the most pronounced effects of endogenous soil nutrient supply in the model. Although such conditions are rare globally, they are prevalent in parts of sub-Saharan Africa, West Asia and South America (Supplementary Fig. 3a and b). For the high-nut scenario, a maximum amount of 500 kg N ha^−1^ a^−1^ was set and the fertilizer was applied automatically by the model based on the plant nutrient deficit in order to virtually eliminate nutrient limitations for plant growth. As shown in Supplementary Fig. 18 and Supplementary Table 4, computed optimal N application rates were mostly <225–280 kg N ha^−1^ a^−1^, which corresponds to present application levels in parts of Europe, the United States of America and China, and values <170 kg N ha^−1^ a^−1^ were found for 50% of all grid cells. Phosphorus was applied at a rigid level of 100 kg P ha^−1^ a^−1^ at planting, which corresponds to common practice in present high-input regions and ensures sufficient P supply for the plant. Although this causes an oversupply in all grid cells, there are no interactions with other soil functions in the model and hence the purpose of eliminating P limitations for plant growth is fulfilled.

### Crop water management scenarios

Irrigation water supply was either turned off in all grid cells in order to mimic rainfed only agriculture or irrigation water was supplied in sufficient amounts. The latter was used for evaluating the effect of soil nutrient contents and soil texture on maize yields alone if water supply from the soil—depending on precipitation patterns and soil hydrologic characteristics—is eliminated as a limiting factor for plant growth. Automatic irrigation takes place in the model if plant water stress limits potential biomass increase by ⩾10% on a given day during the growing season. The model then applies sufficient water to level out plant water stress on this specific day. The total annual allowable amount was set to 2,000 mm in order to fully eliminate water stress.

Supplementary Fig. 3c depicts an example of irrigation volumes applied by the model in scenario high-nut-irr. The actually applied volumes were <440 mm a^−1^ in nearly all grid cells and <155 mm a^−1^ in 50% of all grids (Supplementary Table 4), the highest value found was 1,020 mm a^−1^. Owing to this optimization, the irrigation volumes applied by the model can be considered equal to plant water requirement supplementary to precipitation.

### Model performance evaluation

Model performance in reproducing observed yields around the year 2000, the time period for which reported yields and crop management are representative, was evaluated at the grid cell (Supplementary Fig. 19) and national levels (Supplementary Fig. 20). National average yields based on separate irrigated and rainfed simulations for each grid cell were calculated as


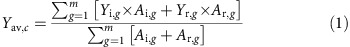


where *Y*_av*,c*_ is the national average yield in country *c*, *Y*_i*,g*_ is yield under irrigated conditions in grid cell *g*, *Y*_r*,g*_ is yield under rainfed conditions in grid cell *g*, *A*_i*,g*_ is irrigated area in grid cell *g* and *A*_r*,g*_ is rainfed area in each grid cell *g*. Average yields per grid cell (Supplementary Fig. 19) were calculate using the same approach without aggregation at the country level. Rainfed and irrigated maize harvest areas per grid cell were adopted form the MIRCA2000 data set^40^. Reported yields were derived from a data set based on national or subnational statistics^41^. The respective grid-cell-specific estimates were then produced for most suitable soils (equation (5)), least suitable soils (equation (6)), dominant soils and soils producing the estimate closest to the reported values.

Among the most suitable, least suitable and dominant soil type, the dominant provides the best correlation with reported yields under present-day reported management conditions at the grid cell level (Supplementary Fig. 19a–c and Table 4). Using the most suitable soil type, results in a lower intercept. When selecting the yield in each grid cell that is closest to the reported value (Supplementary Fig. 19d), *R*^2^ improves significantly and slope and intercept improve slightly. The mean absolute error (MAE) follows a similar pattern (Supplementary Fig. 21a). The magnitude of the MAE is at the grid cell level overall lowest at low-yield levels and highest at high-yield levels. The lowest error occurs for the minimum yielding soil types and soil types with the closest yield subsets at low yields. The lowest error at high yields was found for the latter and the maximum yielding soil types.

Pairwise *t*-tests were applied to compare the MAE of yield estimates between the various subsets of soils, and all the differences were found to be statistically significant (*P*<0.0001). There was not much practical difference in the performance of the models based on most and least suitable soils, respectively, apart from the fact that the former tended to overestimate where the latter tended to underestimate. The estimates of the model, based on the soil producing the yield closest to the observed, understandably had the highest correlation with the observed, smallest MAE and smallest mean squared error (MSE). The validation shows that model performance also strongly depends on which soil type is selected in each grid cell. However, the approach of selecting the soil providing the yield closest to the reported corresponds to a calibration and must be treated with care. It ignores uncertainties originating from management input data like growing seasons, fertilizer inputs, extents of irrigation areas and volumes and planted cultivars as well as uncertainties in model algorithms as, for example, the estimation method for potential evapotranspiration. Also the often coarse resolution of the reported yields data set not only limits the quality of the validation at the grid cell level but would also impact such a calibration approach. It also has to be taken into account that a global validation of crop yields mainly reflects management conditions that vary in the case of maize strongly between countries with high agricultural intensification mainly in the Northern hemisphere and countries with prevailing low-input agriculture.

The agreement between simulated and reported national average yields is far better in terms of statistical coefficients (Supplementary Fig. 20). The maximum yielding soil type causes a systematic overestimation of low yields, which is evident in the high intercept (Supplementary Fig. 20a), whereas the low slope for the lowest yielding soil type is caused by an overestimation of yields in high-yielding countries (Supplementary Fig. 20b). The dominant soil type shows at better agreement in terms of coefficients with reported yields (Supplementary Fig. 20c). Selecting the best matching yield in each grid cell also improves the agreement at the national scale massively (Supplementary Fig. 20d). The MAE at the national scale (Supplementary Fig. 21b) for the minimum yielding soil type follows mostly the pattern at the grid cell level, except for the highest yield bin, in which simulated yields match well. The MAE for the maximum yielding soil type in contrast decreases with increasing reported yields, which is due to the over-estimation of yields in low-yielding countries. The MAEs for the dominant and best matching soil types show a similar pattern with constantly comparably low errors.

### Yield estimates across time and soil types

Different subsets of crop yield estimates were analysed to identify and compare different sources of variability. The whole set of possible yields within a grid cell is represented as


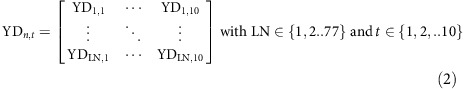


where (YD_*n,t*_) is the crop yield on a given soil type *n* in year *t* and LN describes the number of soil types in a given grid cell. These can range between 1 and 77. *n* also identifies the ranking of soil types according to their aerial coverage with *n*=1 standing for the dominant soil type. *t* refers to the simulation years and ranges from 1 to 10. Hence,


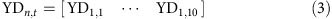


describes the annual yields on the dominant soil type (*n=1*) in each grid cell and


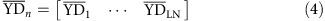


refers to the 10-year means of yields across all soil types (*n*) up to the number of soil types (LN) in each grid cell.

### Identification of most and least suitable soils

The most or least suitable soil types were defined as maximum or minimum yielding soil types within a grid cell. They were identified by moving through the yield estimates for each grid cell from the dominant to the least abundant soil and selecting the soil type providing the highest or lowest yield according to


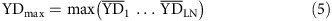


and


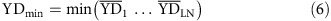


where YD_*n*_ is the array of 10-year mean yields for all soil types in a grid as shown in equation (4).

### Arithmetic and area-weighted coefficient of variation

The coefficient of variation (CV, %) as a measure for yield variability was calculated as


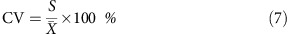


where *S* is the standard deviation and 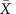 is the mean of a sample. Thereby, the combination of equation (7) and the yield sample in equation (2) is termed CV_tot_, the combination of equation (7) and yield sample in equation (3) is CV_dom_, and the combination of equation (7) and the yield sample in equation (4) is CV_soil_.

An area-weighted CV_aw_ based on the CVs analogue to the yields in equation (4) was calculated as


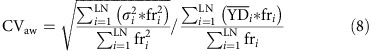


where *σ*_*i*_ is the standard deviation of yields on soil type *i*, fr_*i*_ is the fraction of coverage by soil type *i*, YD_*i*_ is the mean yield for soil type *i* and LN is the number of soil types in the respective grid cell.

### Statistical evaluation

The statistical significance of differences between yield subsets in Fig. 2 was tested by an analysis of variance combined with Tukey's honest significant difference (HSD) test at *P*=0.05. Tukey's HSD tests whether the means of two samples are significantly different from each other, which is indicated by different letters. Yields were log-transformed for both tests to achieve a normal distribution. Details of the evaluation results are provided in Supplementary Table 2. Kendall's tau-test in a modified form that accepts ties within a sample was used for evaluating the correlation between the ratios of CV_tot_ for the bau-nut and no-nut management scenarios and fertilizer application rates in various climate regions (Fig. 3 and Supplementary Table 3). This test evaluates a rank correlation between two non-parametric samples, which is provided if *tau*≠0 and *P*<0.01.

### Relevant routines of the EPIC model

The subsequently outlined routines are an excerpt of the model processes relevant for interpreting the results presented herein. Their descriptions are based on the original documentation of the EPIC model, which has been made publicly available by the developers at http://epicapex.tamu.edu/files/2015/05/EpicModelDocumentation.pdf.

Phenologic development of the crop takes place according to the heat unit (HU) approach. Daily HU are calculated as


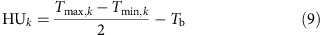


where HU_*k*_ is the HUs accumulated on day *k* [°C], *T*_max*,k*_ and *T*_min*,k*_ are the maximum and minimum temperatures on the day (°C) and *T*_b_ is the base temperature (°C) of a specific crop. Maturity is reached when total accumulated HU are equal to potential HUs (°C), the sum of the daily HU based on long-term climate data and reported growing seasons provided by Sacks *et al*.^38^

EPIC estimates potential biomass increase Δ*B*_p_ on each day according to


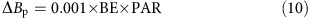


where Δ*B*_*p*_ (t ha^−1^) is biomass gain, BE [(kg ha^−1^)/(MJ m^−2^)] is the biomass-energy-conversion coefficient and PAR [MJ m^−2^] is intercepted photosynthetic active radiation depending on leaf area index (LAI) and solar radiation. Actual biomass is subsequently obtained by correcting Δ*B*_p_ for the maximum stress out of nutrients, water, temperature, aeration, salinity (see further below).

At maturity, crop yield is calculated by multiplying total above-ground biomass with a water stress-adjusted harvest index (HIA^*^). Many grains like maize are most sensitive to water stress during flowering, when major yield components are determined^42^ and barrenness of flowers can cause massive yield losses^43^. The water-stress adjusted HI (HIA*) is estimated from simulated potential HI (HIA; depending on HU accumulation) and a defined minimum HI (HIA_*0*_) according to


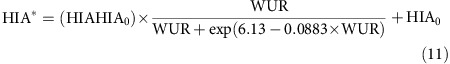


where WUR is the simulated water use ratio. WUR is estimated at harvest as


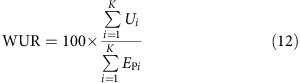


where *U*_*i*_ [mm d^−1^] is the actual and *E*_P*i*_ [mm d^−1^] the potential plant water use rate for day *i*. *K* is the total number of days of the growing season. The exponential parameters in equation (11) are set to result in 0.05 × (HIA-HIA_0_) when WUR=0.10 and 0.90 × (HIA-HIA_0_) when WUR=0.50. Hence, there is little reduction in HIA^*^ if the ratio of *U* to *E*_P_ is greater than 0.5.

Above-ground biomass growth is constrained mainly by water, nutrients (N and P), temperature and aeration stress. The major stress on each day of the growing season limits biomass accumulation by a fraction ranging from 0 to 1. The sum of the daily values for each stress factor over the growing season is referred to as ‘stress days'. The stresses are computed as follows:

The effect of water deficit or water stress (WS) on plant biomass production is based on the concept that drought stress is proportional to the transpiration reduction^44^. It is calculated as


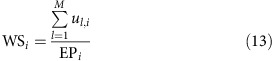


where WS_*i*_ is the amount of water stress on day *i* [−], *l* is a given soil layer [−], *M* is the total number of soil layers [−], *u*_*l,i*_ is the plant available water in layer *l* on day *i* [mm], and EP_*i*_ is the potential ET on day *i* (mm). In addition, water deficit has an impact on HI as described above. Temperature stress (TS) occurs on a given day *i* if the average air temperature TG is above the optimum temperature (TO) or below the base temperature (TB) according to


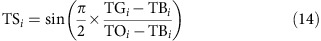


or if the average daily temperature exceeds TO by 50%. Nutrient stresses (N stress (NS) and P stress (PS)) vary nonlinearly between optimum or excessive supply and 50% of the optimum supply when stress is 100% (ref. 45). First, a scaling factor SNS (here for *NS*) on a given day *i* is calculated as


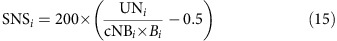


where UN_*i*_ is the N uptake on day *i* (kg ha^−1^), cNB_*i*_ is the optimum N concentration in biomass on day *i* (kg kg^−1^) and *B*_*i*_ is the total plant biomass on day *i* (kg ha^−1^). This factor is then used for estimating the actual nitrogen stress NS according to


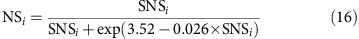


The calculation of PS follows the same pattern. Aeration stress occurs if the soil pore space approaches water saturation. A crop-specific saturation factor SAT for day *i* is estimated as


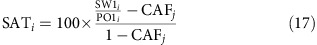


where SW1 is the soil water content (mm) on day *i* in the top 1 m of the soil profile, PO1 is the pore volume (mm) on day *i* in the top 1 m of the soil profile, and CAF is the critical aeration factor [−] of crop *j*, which can vary between 0 and 1. If SAT_*i*_>0, aeration stress (AS) is subsequently calculated as


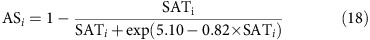


Further plant growth constraints occur through limitations of root growth, whereas EPIC selects that major stresses out of soil strength, aluminium toxicity and temperature stress to limit root development on a given day. Soil strength depends on bulk density according to


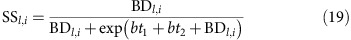


where SS_*l,i*_ is the soil strength [−] of layer *l* on day *i*, BD_*l,i*_ is the bulk density (g cm^−3^) of layer *l* on day *i* and *bt*_1_ and *bt*_2_ are coefficients based on the soils sand content. Aluminium toxicity stress (ATS) depends on the amount of aluminium set free at a certain pH and the aluminium sensitivity of the crop. The amount of aluminium (AL0) a crop *j* can tolerate is estimated according to


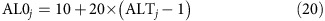


where ALT_*j*_ is an aluminium tolerance index that can vary between 1 (=highly sensitive) and 5 (=very tolerant). ATS on day *i* is then estimated as


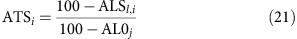


where ALS_*l,i*_ is the amount of dissolved aluminium in soil layer *l* on day *i*.

SOM and organic nitrogen cycling follow the approach of the CENTURY model^46^. As described in Izaurralde *et al*.^47^, SOM is split into several pools with varying exchange and turnover rates: standing dead residue and roots, metabolic and structural litter, slow humus, passive humus and microbial biomass. C, N and P may leave the system through erosion, leaching and volatilization. Fluxes between different pools depend on soil and crop management, soil hydrology, temperature and depth within the profile. The C/N ratio has in addition an impact if microbial processes are involved.

Out of various options for calculating water erosion in EPIC, the MUSLE approach was selected as it has been adapted to small watersheds, which have most similarity with single agricultural fields. The algorithm is based on rainfall kinetic energy, soil erodibility, crop management, erosion control practice, slope length and steepness, and soil coarse fragment content. Daily wind erosion is estimated taking into account soil erodibility, surface roughness, vegetative cover, mean unsheltered travel distance of wind across the field and duration of wind greater than threshold velocity (here with a default of 6 m s^−1^). Atmospheric deposition as an opposite process was not taken into account. Recent studies have shown that atmospheric loading with and deposition of N and P strongly depend on emissions from fossil fuel combustion and atmospheric transport processes^48^,^49^,^50^. Resulting deposition rates are presently not available in a form suitable for global crop models, but should be included in the future.

The Hargreaves method^51^ was used for calculating potential evapotranspiration. Potential soil evaporation depends on potential evapotranspiration and soil cover. Actual soil evaporation was estimated from the top 20 cm of the soil profile.

## Additional information

**How to cite this article:** Folberth, C. *et al*. Uncertainty in soil data can outweigh climate impact signals in global crop yield simulations. *Nat. Commun.* 7:11872 doi: 10.1038/ncomms11872 (2016).

## Supplementary Material

Supplementary Information Supplementary Figures 1-21, Supplementary Tables 1-4 and Supplementary Discussion.

## Figures and Tables

**Table 1:** Percentage of grid cells with CV_tot_ higher than CV_dom_.

Management*	**Rainfed**	**Irrigated**
	**CV**_**tot**_**>CV**_**dom**_	**CV**_**tot**_**>CV**_**dom**_
No-nut	89.7	96.1
Bau-nut	76.7	75.4
High-nut	61.5	28.7

*Bau-nut, business-as-usual nutrient inputs; CV, coefficient of variation; High-nut, sufficient nutrient inputs; No-nut, no nutrient inputs.

**Table 2:** Percentages of grid cells in which yield variability is largest for CV_soil_, CV_dom_ or not dominated by either one as displayed in Fig. 4.

Management*	**Rainfed**			**Irrigated**		
	**Soil**	**Climate/soil**	**Climate**	**Soil**	**Climate/soil**	**Climate**
No-nut	50.6	25.7	23.7	81.0	13.0	6.0
Bau-nut	33.0	24.5	42.6	45.4	19.4	35.3
High-nut	17.7	17.9	64.5	1.4	4.4	94.3

*Bau-nut, business-as-usual nutrient inputs; CV, coefficient of variation; High-nut, sufficient nutrient inputs; No-nut, no nutrient inputs.

**Table 3:** Nutrient and water management scenarios used in the study.

Fertilizer scenario*	**Abbreviation**	**Max. irrigation vol. (mm a**^−**1**^)	**N (kg ha**^−**1**^** a**^−**1**^)	**P (kg ha**^−**1**^** a**^−**1**^)
Business as usual	Bau-nut-irr	2,000*	Variable†	Variable†
Business as usual	Bau-nut-rf	—	Variable†	Variable†
No nutrient inputs	No-nut-irr	2,000*	—	—
No nutrient inputs	No-nut-rf	—	—	—
Sufficient nutrients	High-nut-irr	2,000*	Max. 500	100
Sufficient nutrients	High-nut-rf	—	Max. 500	100

^*^The upper limit of irrigation assumes sufficient supply of irrigation water, which is presently the norm in global crop simulations 6 due to lack of data for spatial crop-specific application volumes.

^†^Business as usual refers to fertilizer application rates around the year 2000 (see the Methods for details).

**Table 4:** Evaluation of model performance for various soil subsets at the grid cell level.

**Soil subset**	***r***^**2**^	**ME**	**MSE**	**MAE**
Most suitable	0.29 (*P*<.0001)	0.46	6.07	1.87
Least suitable	0.29 (*P*<.0001)	−1.16	6.52	1.76
Dominant	0.36 (*P*<.0001)	−0.25	4.93	1.55
Closest to the observed	0.54 (*P*<.0001)	−0.40	3.34	1.07

MAE, mean absolute error; ME, mean error; MSE, mean squared error.
