# Supplementary material for: Uncertainty in soil data can outweigh climate impact signals in global crop yield simulations
Source: Nat Commun. 2016 Jun 21;7:11872. doi: 10.1038/ncomms11872 (PMC4919520; doi:10.1038/ncomms11872)
Supplement: Supplementary Information — Supplementary Figures 1-21, Supplementary Tables 1-4 and Supplementary Discussion. [file ncomms11872-s1.pdf]

**Supplementary Information** for *"Uncertainty in soil data can outweigh climate impact signals in global crop yield simulations"*

*Authors: C Folberth, R Skalský, E Moltchanova, J Balkovič, L Azevedo, M Obersteiner, M van der Velde*

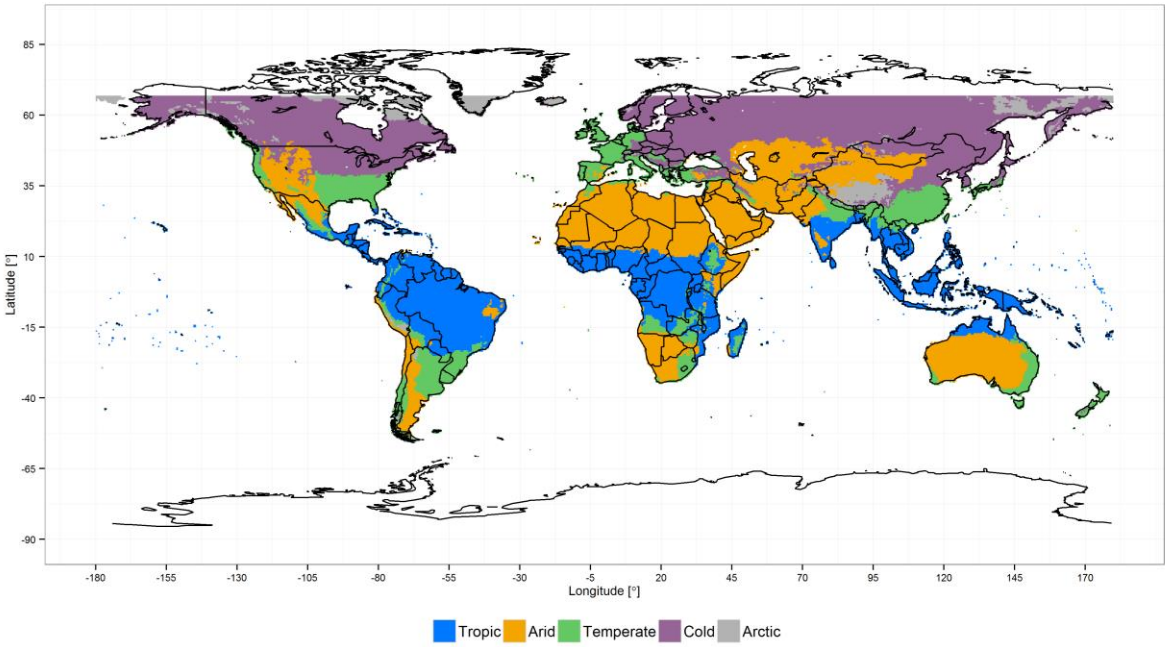


**Supplementary Figure 1: Major Koeppen-Geiger climate regions.** Grid cells above 67°N are presently not cultivated and were not taken into account for climate data processing. The rules for defining the climate regions are listed in Supplementary Table 1.

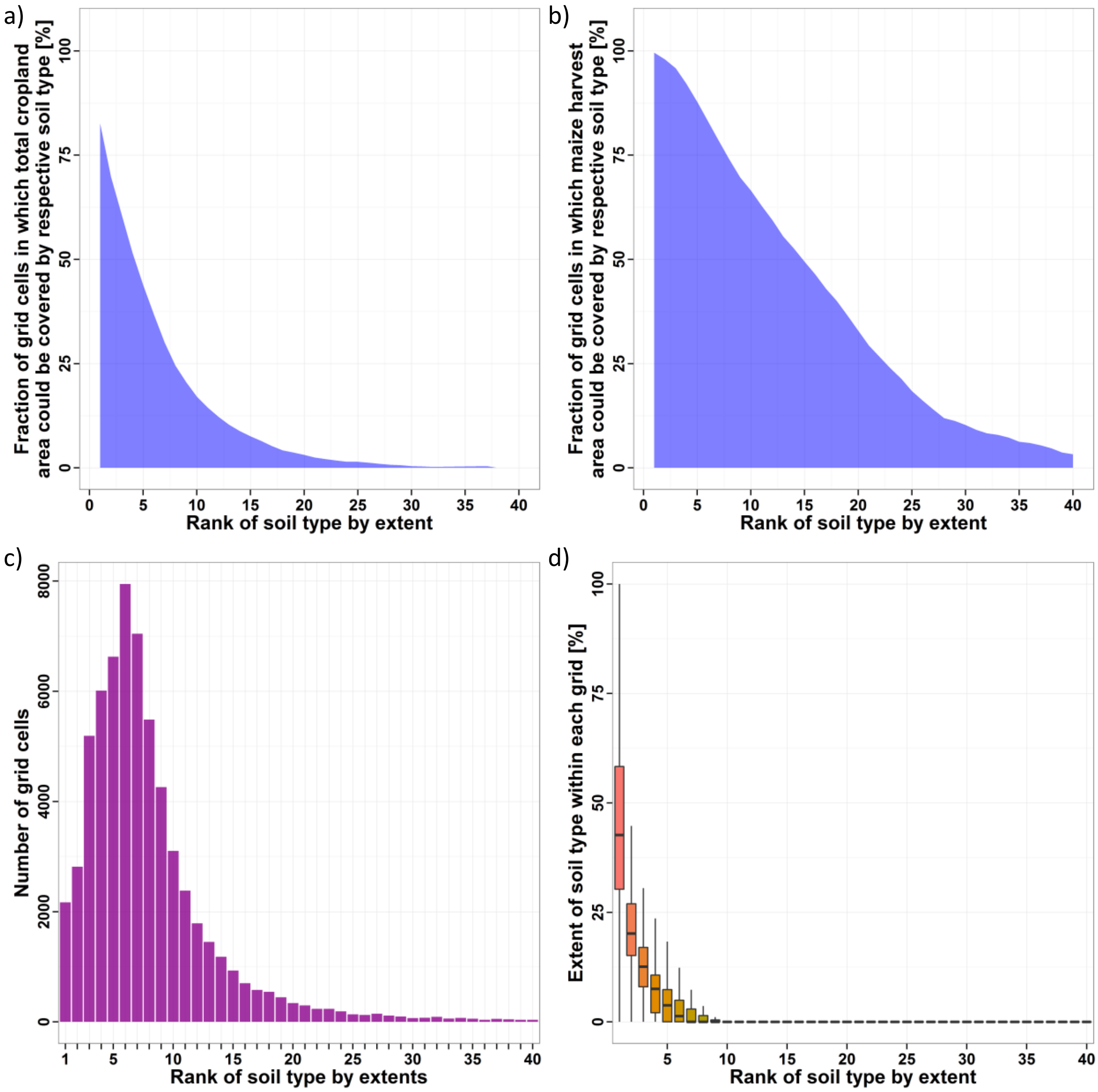


**Supplementary Figure 2: Fraction of grid cells in which (a) all cropland or (b) present maize harvested area could be covered by each respective soil type in the HWSD. This refers only to grid cells that contain the respective soil type. (c) Maximum number of soil types per grid cell and (d) ranges of extents of soil types in each grid.** Grid cells with more than 40 soil types have been excluded for better readability.

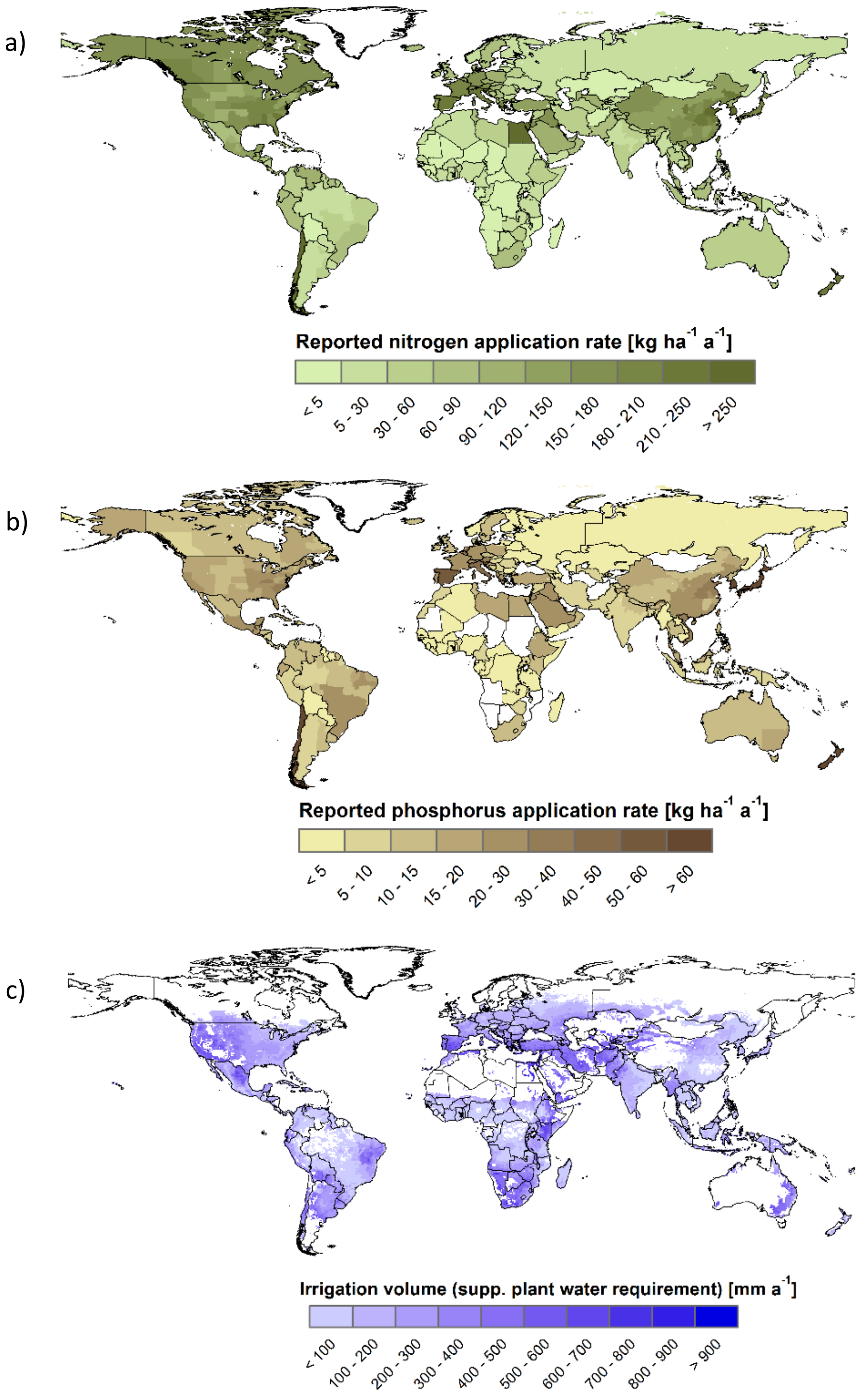


**Supplementary Figure 3: (a) Nitrogen and (b) phosphorus fertilizer application rates as provided by Elliott *et al.*[1]. (c) Simulated irrigation volumes estimated by the model using automatic water application if water stress exceeds 10% growth limitations on a given day.**

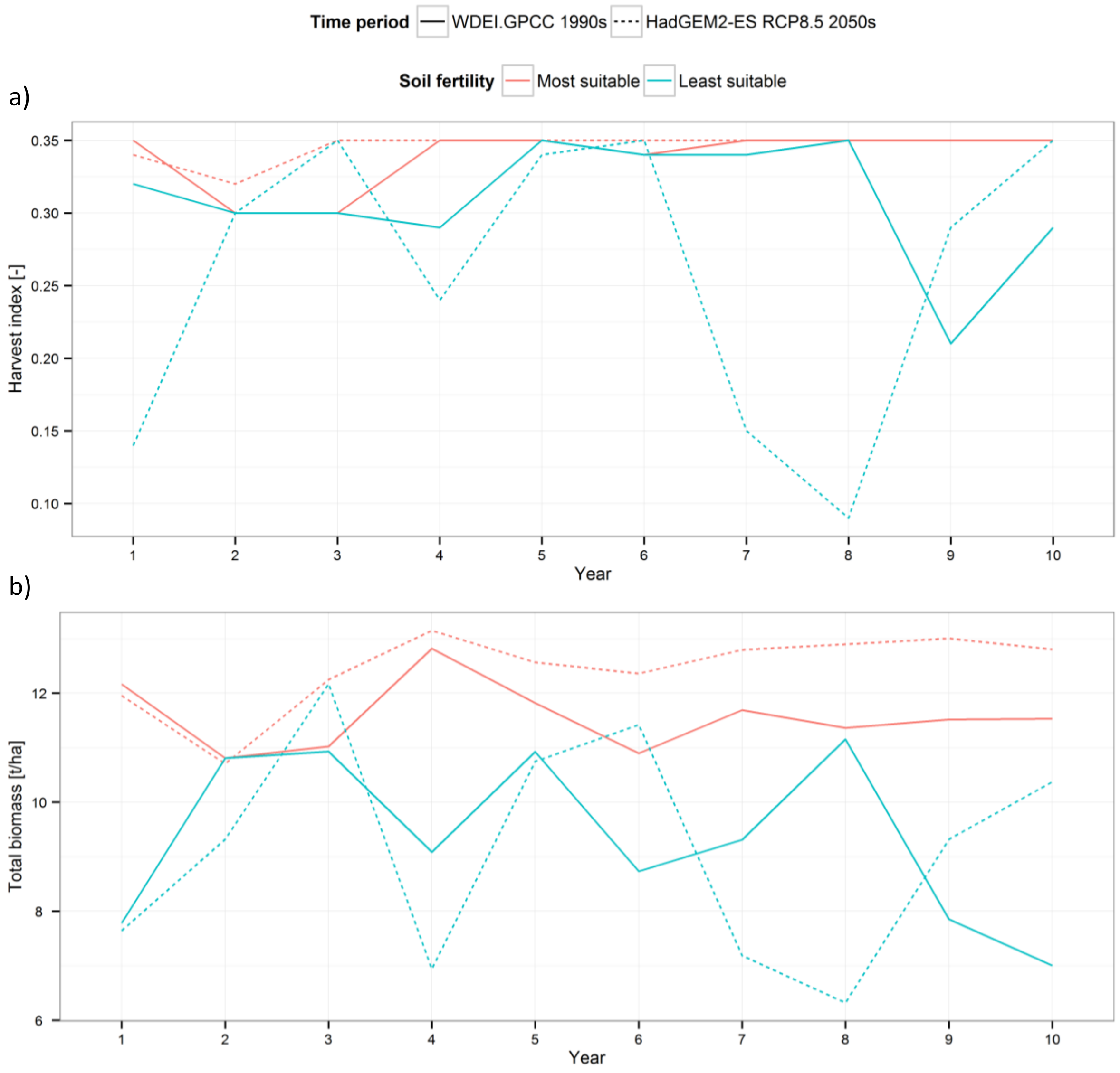


**Supplementary Figure 4: Annual (a) harvest index and (b) total biomass on the most and least suitable soil types during an evaluation period of ten years under historic or potential future climate.**

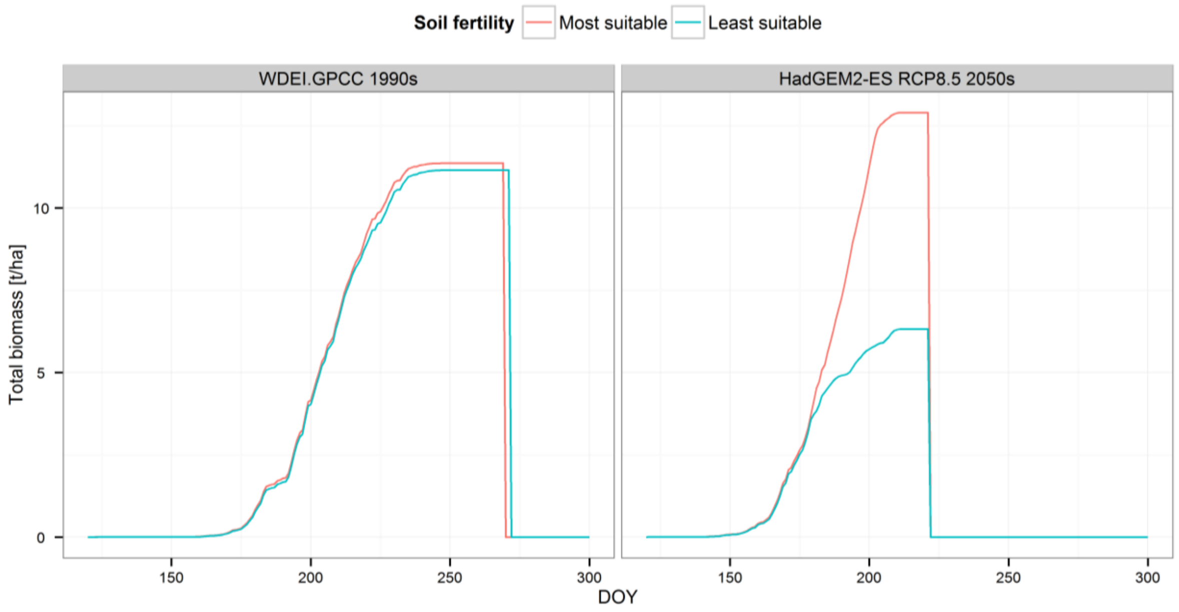


**Supplementary Figure 5: Evolution of total biomass during the historic and projected future time period on the most and least suitable soil types in the 8th simulation year.**

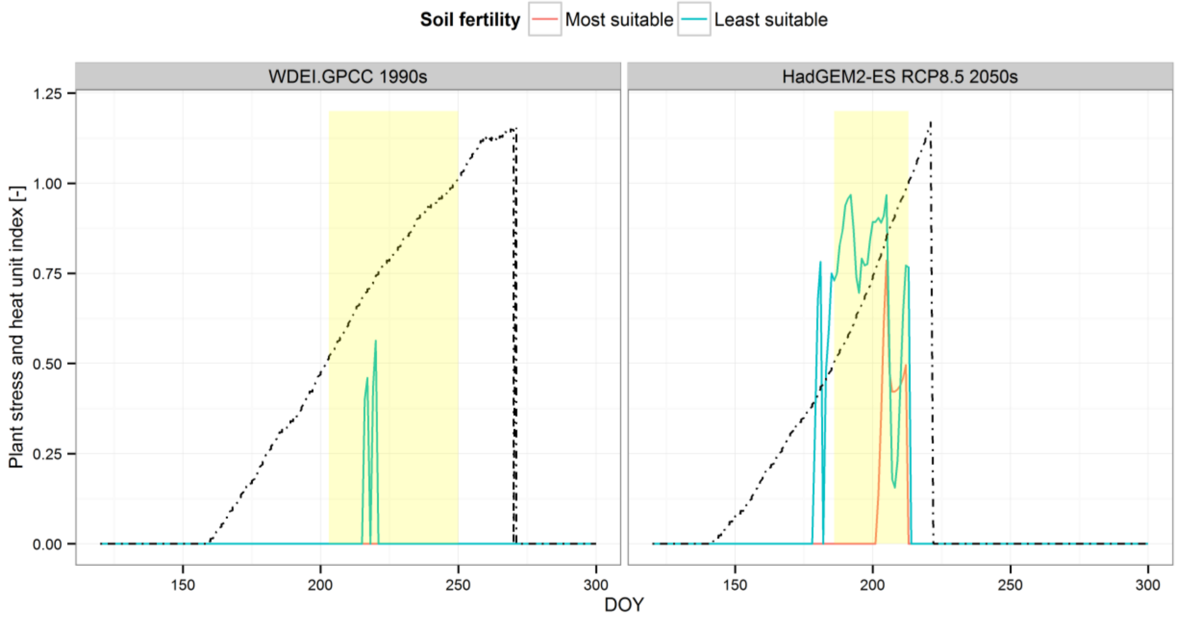


**Supplementary Figure 6: Plant water stress and heat unit index during the historic and projected future time period on the most and least suitable soil types. The yellow area marks the second half of the growing season, which is the period of flowering.** Stress factors are a fraction between 0-1 [-] that is used to limit potential biomass increase in model on each day (see equation (13)-(21) in Methods section). The heat unit index (HUI; dashed line) shows accumulated heat units. Plant maturity is reached when HUI=1 and harvest takes place at HUI=1.15, which takes post-maturity drying of the crop on the field into account.

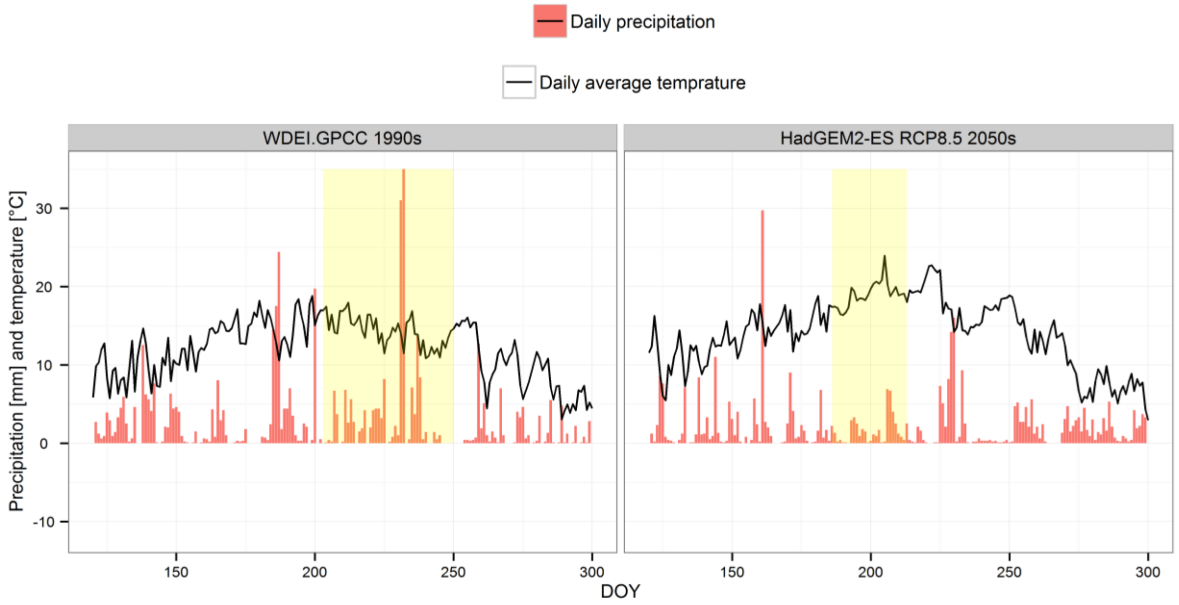


**Supplementary Figure 7: Daily average temperature and precipitation during the historic and projected future time period on the most and least suitable soil types.** The yellow area marks the second half of the growing season, which is the period of flowering.

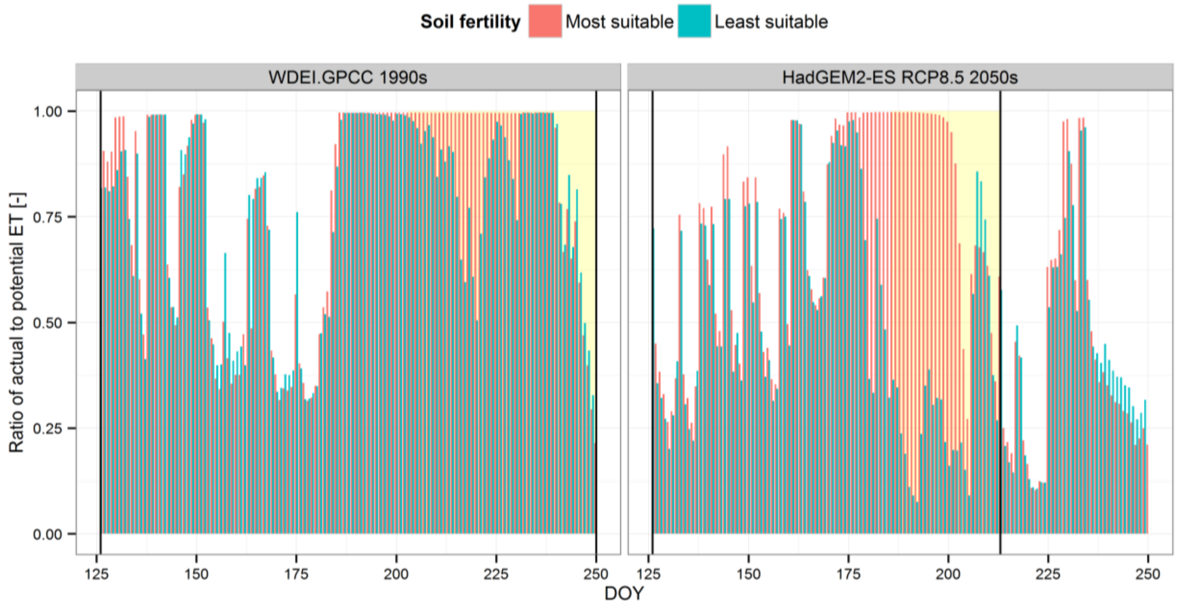


**Supplementary Figure 8: Ratio of actual to potential ET during the historic and projected future time period on the most and least suitable soil types.** Vertical black bars indicate the beginning and end of the growing season during which the ration of actual to potential ET corresponds to the water use ratio (WUR; see equation (12) in Methods section). The yellow area marks the second half of the growing season, which is the period of flowering. Yield formation is limited by adjusting the harvest index whenever the ratio is <0.5.

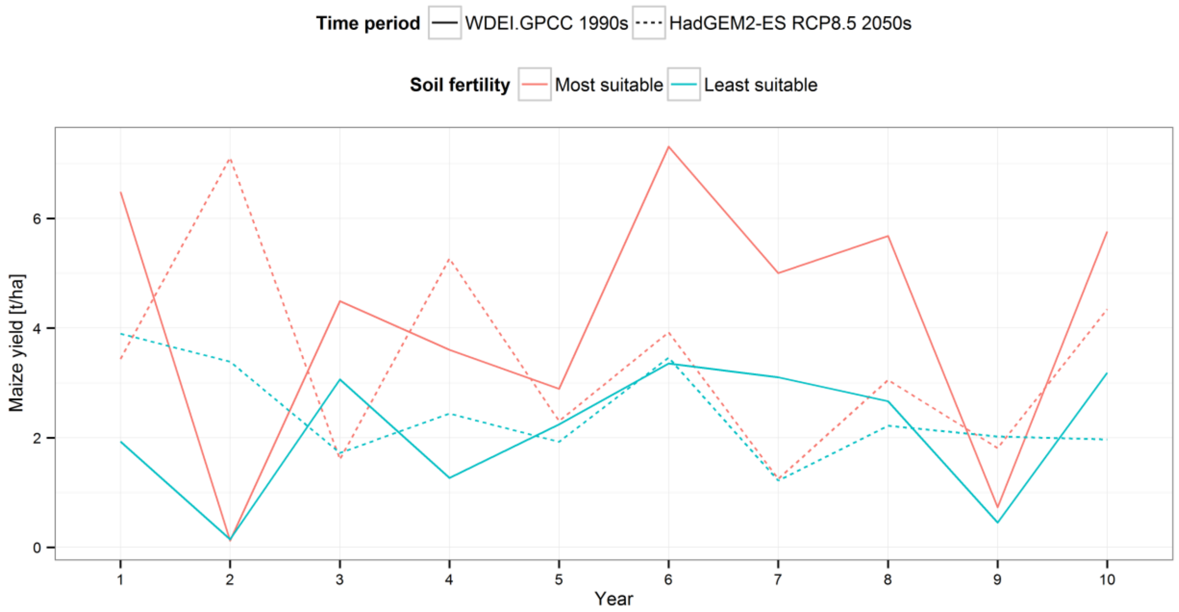


**Supplementary Figure 9: Annual maize yields on the most and least suitable soil types during an evaluation period of ten years under historic or potential future climate.**

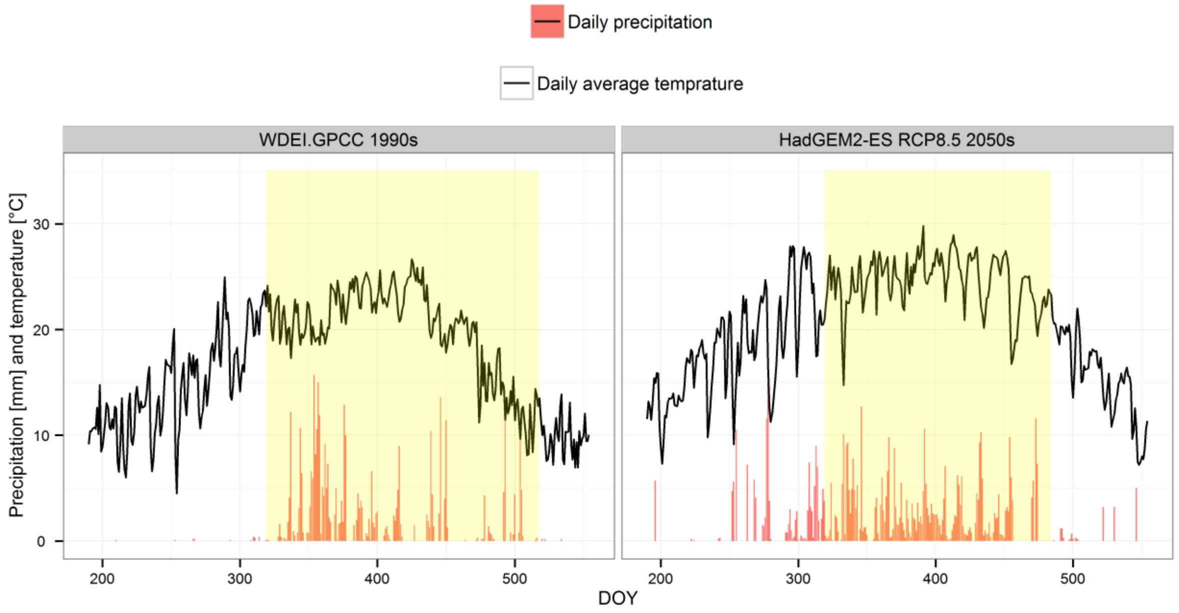


**Supplementary Figure 10: Daily average temperature and precipitation during the historic and projected future time period on the most and least suitable soil types.** The yellow area marks the whole growing season.

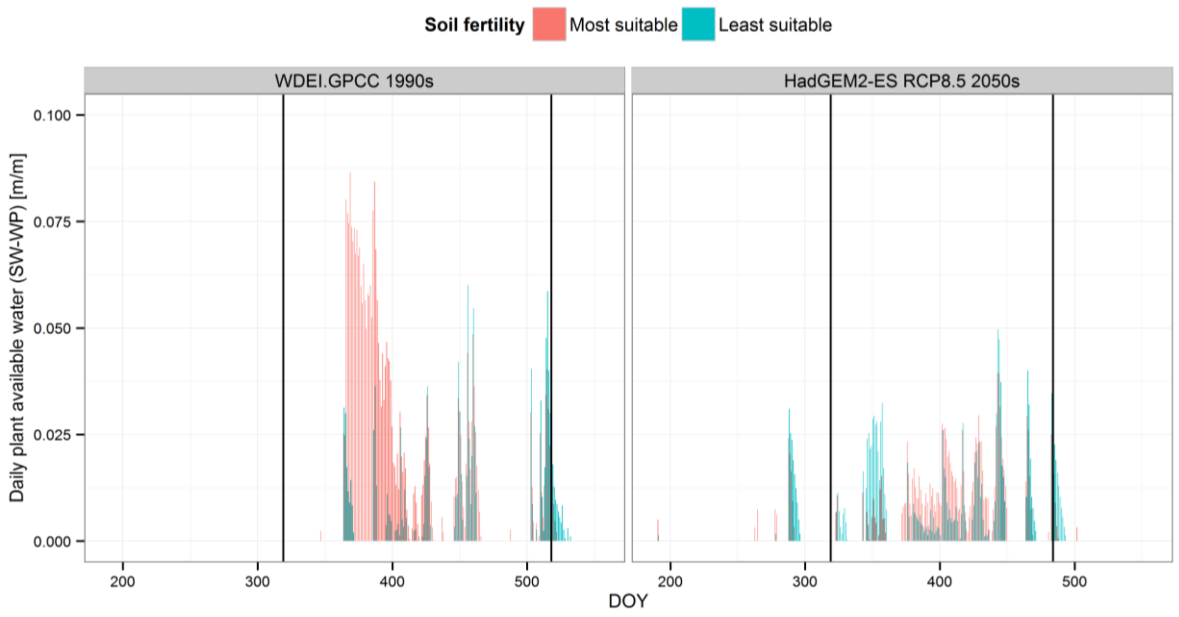


**Supplementary Figure 11: Daily plant available water during the historic and projected future time period on the most and least suitable soil types. The vertical lines mark the growing season.** Plant available water is calculated as [soil water content] – [water content at wilting point].

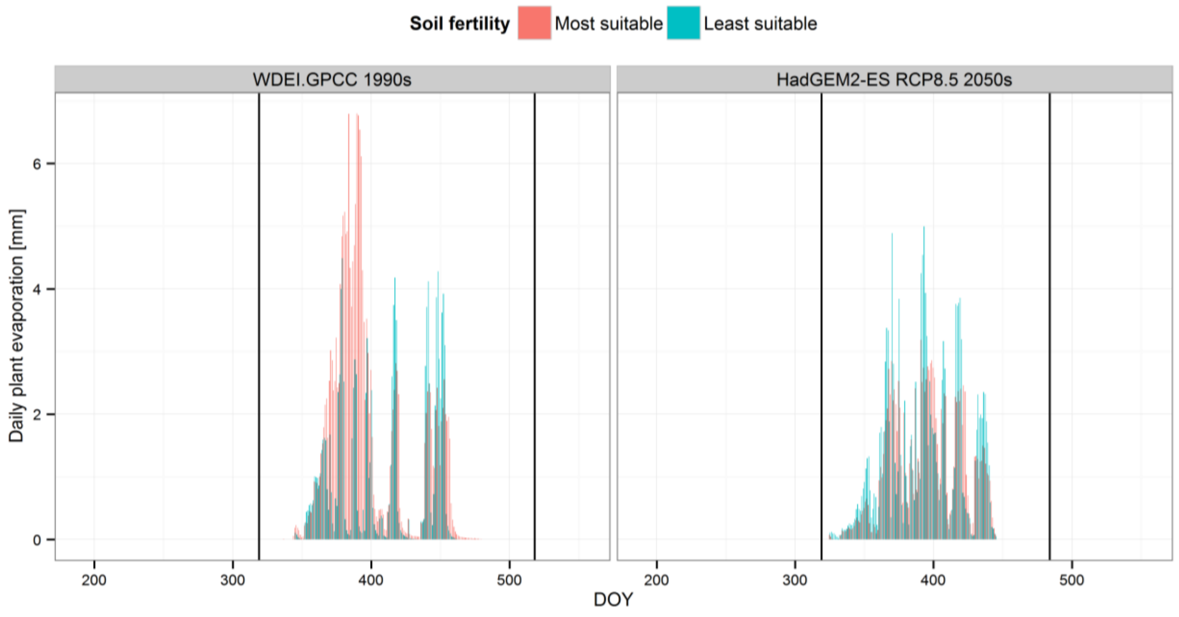


**Supplementary Figure 12: Daily plant evaporation during the historic and projected future time period on the most and least suitable soils.** The vertical lines mark the growing season.

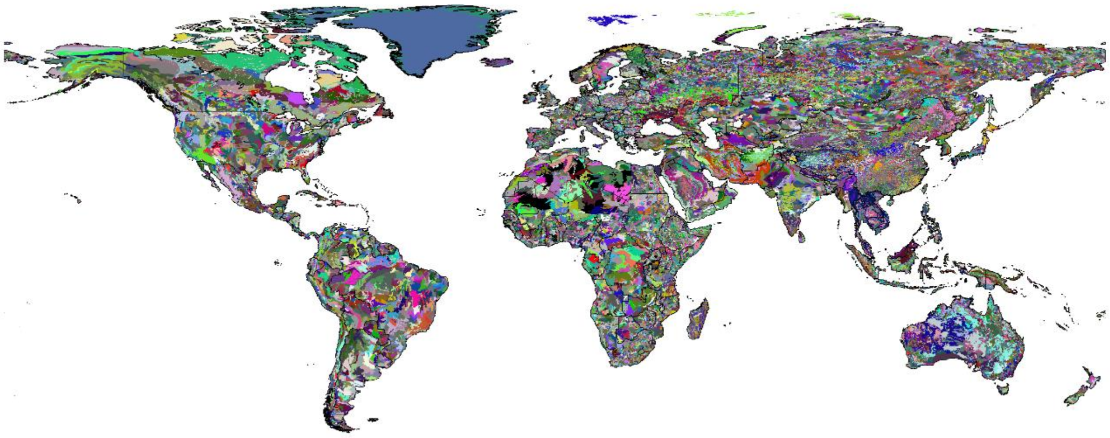

**Supplementary Figure 13: Soil mapping units (SMUs) and their extent in the HWSD version 1.2.** Colors serve solely for delineating SMUs. Large extents of SMUs can be found especially in arctic and arid regions, where soil variability is low and soil texture is strongly dominated by either rocks or sand or the area is permanently covered by ice. SMUs with very small extents have been reported for parts of Central Europe, East Africa and especially China (see Supplementary Fig. 17a).

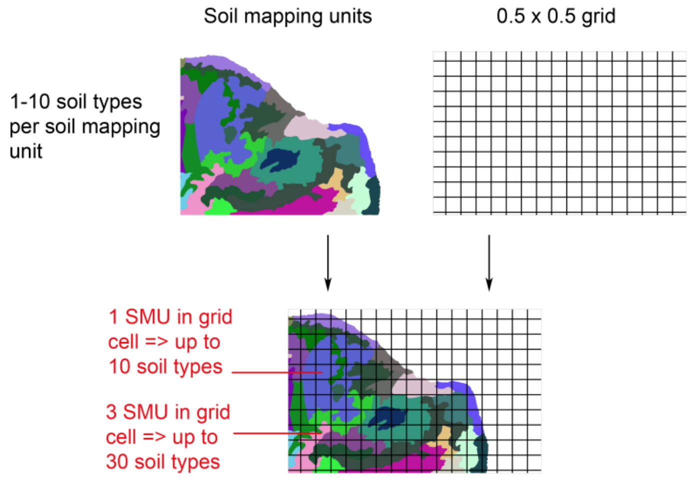


**Supplementary Figure 14: Aggregation of soil mapping units to 0.5° x 0.5° resolution.** The original soil map is based on polygons with varying sizes (e.g. very fine resolution in China, coarse resolution in Central Africa). Each of the polygons links to data for 1-10 soil types including their extents. When transforming the soil map to a 0.5° x 0.5° grid, this results in up to 77 soil types.

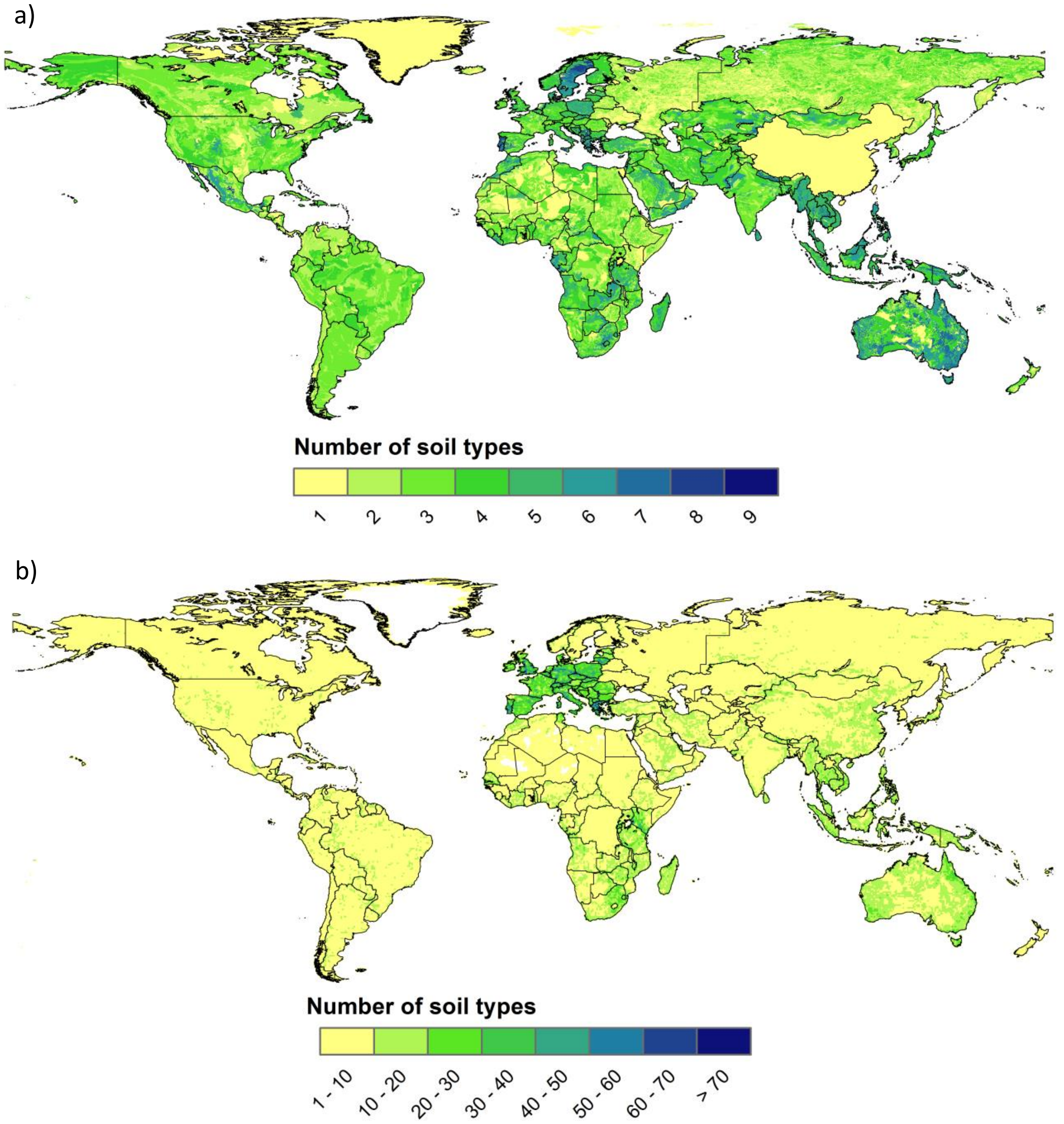


**Supplementary Figure 15: Number of soil types for (a) original SMUs at a resolution of 30 arc seconds and (b) aggregated to a grid of 0.5° x 0.5° resolution.** The number of soils types per SMU in the original dataset (panel a) is highest in Scandinavia, Eastern Europe, Southeast Asia, and Australia and low in Western Russia, the Central USA and the Sahel, while only one soil type is reported for each SMU in China and Greenland. After aggregation to 0.5°x0.5° (panel b) the largest number of soil types per grid cell can be found in Europe, Southeast Asia, East Africa and Australia, moderate numbers in China, West Asia, and Western Africa. In the case of China, this is due to the very high spatial resolution of SMUs in most parts of the country (Supplementary Fig. 17a).

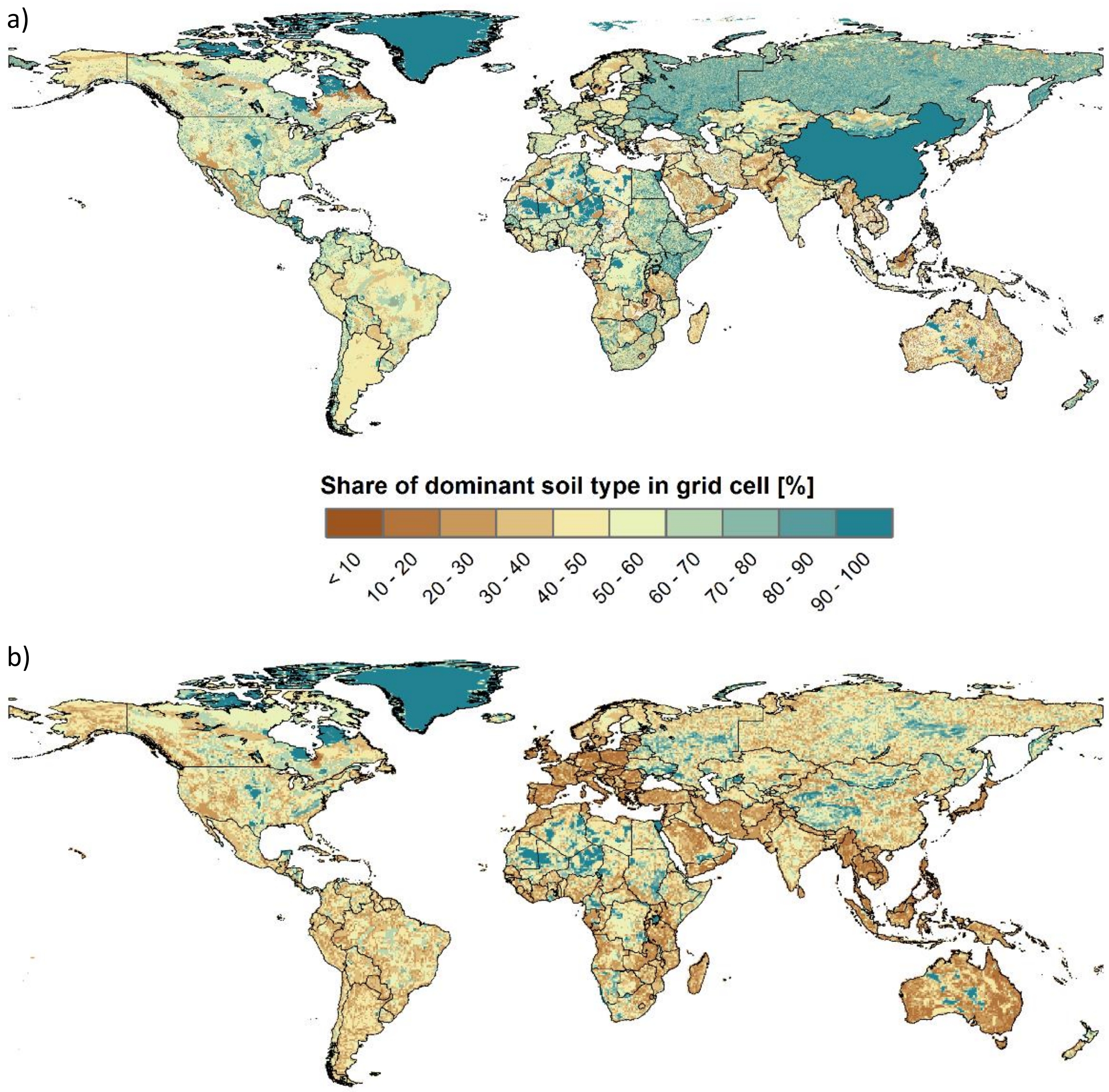


**Supplementary Figure 16: Share of the dominant soil type for (a) original SMUs at a resolution of 30 arc seconds and (b) aggregated to a grid of 0.5° x 0.5° resolution.** In all grid cells or SMUs for which only one soil type has been reported, the share of the dominant soil type is 100% in the native dataset (panel a). These are all of China and Greenland, and parts of the Sahara, central Australia, and North America, where sand and rock are dominating. Also most parts of the former Soviet Union and Northeast Africa show large shares for the dominant soil type. After aggregation to a 0.5° x 0.5° resolution (panel b), the share of the dominant soil type decreases massively in most of these regions except for Greenland, Northeastern Canada, central Australia and the Sahara, due to the small sizes of SMUs.

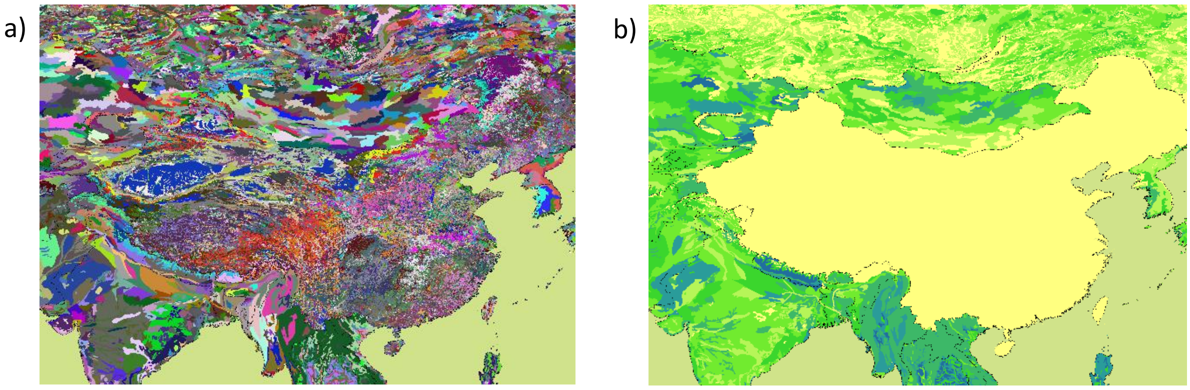


**Supplementary Figure 17: (a) Soil mapping units (SMU) in East Asia and (b) number of soil types per SMU in the same region.** Colors in panel (a) serve solely for delineating the various SMUs, the scale in panel (b) corresponds to that of Supplementary Fig. 15a.

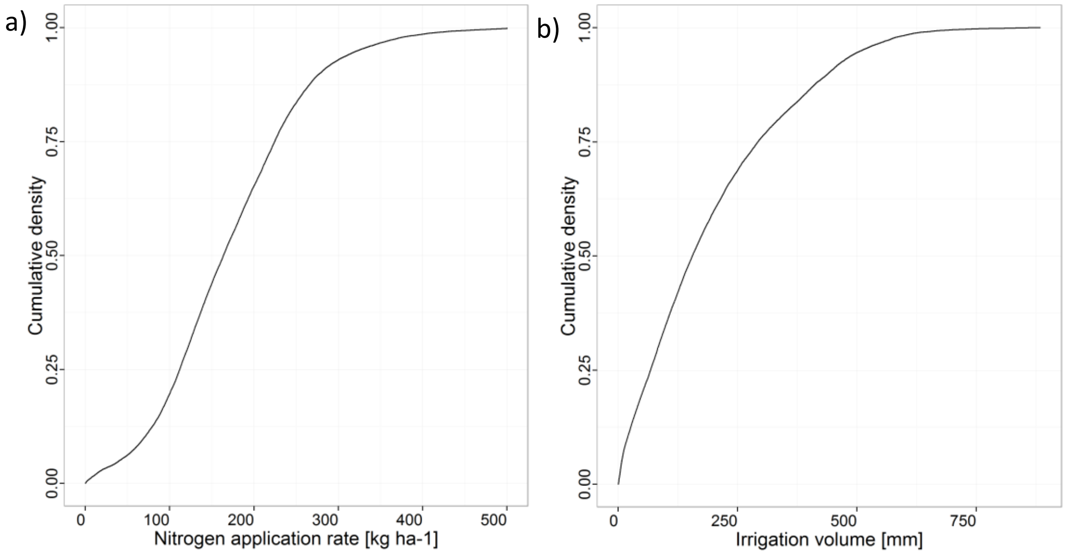


**Supplementary Figure 18: Cumulative density of (a) N application rates and (b) irrigation volume for the dominant soil type in scenario high-nut-irr (see Table 3 in the article).**

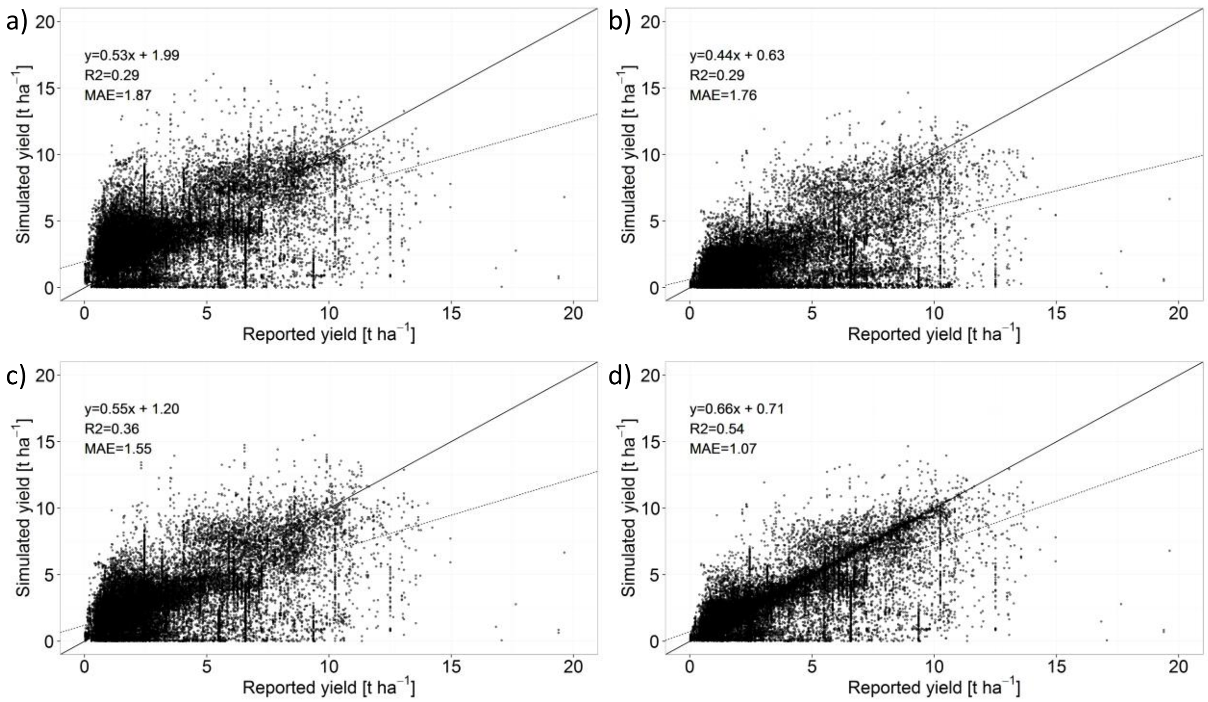


**Supplementary Figure 19: Validation of model performance at the grid cell level for the (a) most suitable soil type, (b) least suitable soil type, (c) dominant soil type, and (d) soil type providing the yield closest to the value reported by Monfreda *et al.*[2].** Rainfed and irrigated yields for the bau-nut scenario were weighted in each grid cell according to the MIRCA2000 dataset[3]. MAE=mean absolute error.

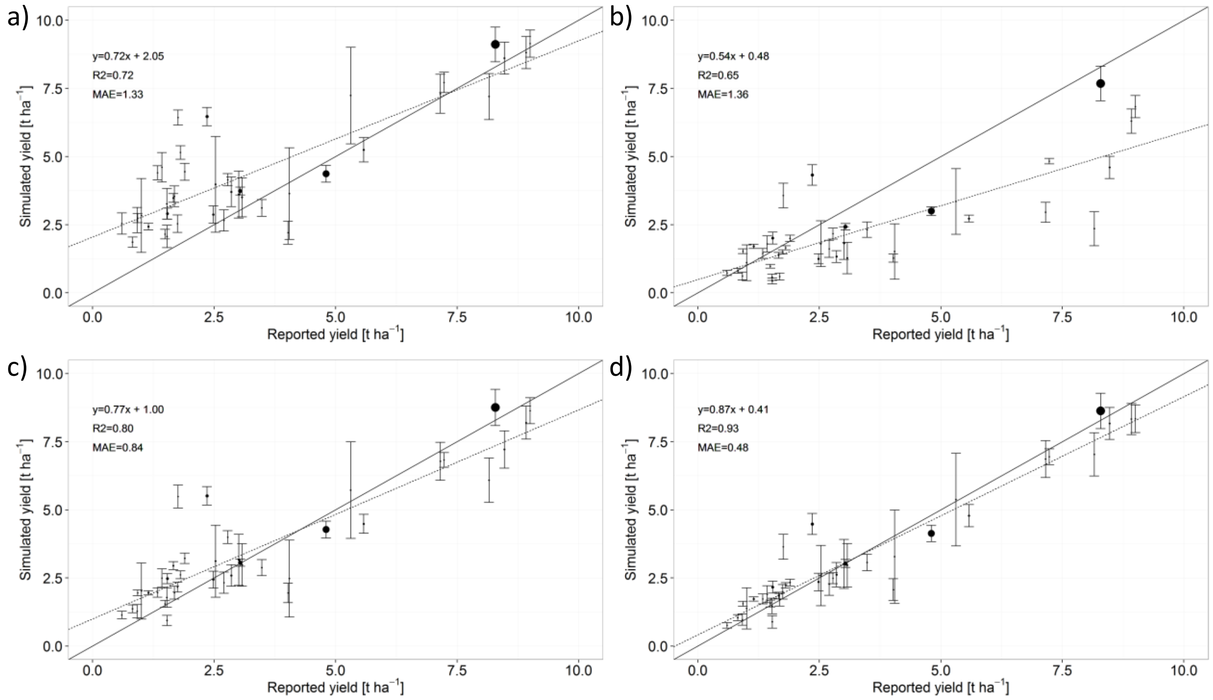


**Supplementary Figure 20: Validation of model performance at the country level for (a) the most suitable soil type, (b) least suitable soil type, (c) dominant soil type, and (d) soil type providing the yield closest to the value reported by Monfreda *et al.*[2].** Rainfed and irrigated yields for the bau-nut scenario were weighted in each grid cell according to the MIRCA2000 dataset[3]. Point size reflects the extent of harvested area within each country error bars show one standard deviation. Results are shown for the 40 major maize producing countries comprising >90% of the total maize harvest area around 2000.

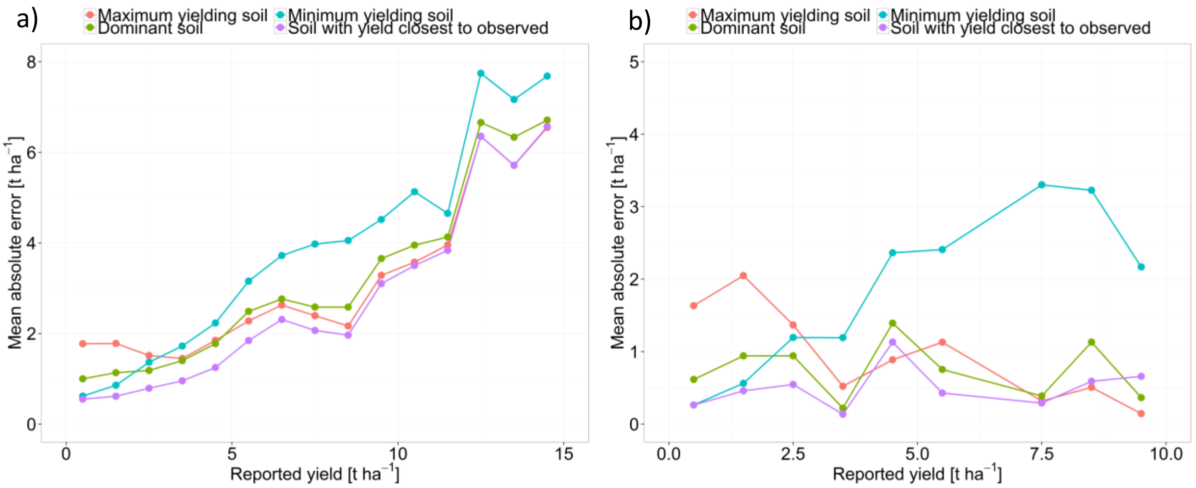


**Supplementary Figure 21: Mean absolute error (MAE) for yield bins of 1 t ha$^{-1}$ at (a) the grid cell level (corresponding to Supplementary Fig. 19) and (b) the national level (corresponding to Supplementary Fig. 20).** In the yield bin of 4-5 t ha$^{-1}$ of panel (b), the MAE for the best matching soil type is higher than that of the maximum yielding soil type. This is caused by a spatial disagreement of high and low-yielding grid cells in Thailand: the best matching yields in some grid cells are still lower than the reported values, while the maximum yielding soil types in other grids overestimate reported yields. Due to the national averaging, the national yield of the highest yielding soil type is hence closer to the reported national average. This adds to the aforementioned limitations of calibrating yields by a single variable and emphasizes the limitations of input and validation data for assessing model performance in detail at the global scale.

**Supplementary Table 1: Climate envelopes used for defining major Koeppen-Geiger climate regions as shown in Supplementary Fig. 1 based on the rules by Peel *et al*.[4].**

| **Name** | **Climate envelope** |
|---|---|
| Arctic | T in hottest month ≤ 10°C |
| Arid | Precipitation < potential ET |
| Cold | T in hottest month > 10°C; T in coldest month ≤ 0°C |
| Temperate | T in hottest month > 10°C; T in coldest month > 0°C and < 18°C |
| Tropic | T in coldest month ≥ 18°C |

**Supplementary Table 2: Statistical coefficients for ANOVA and Tukey's honest significant difference (HSD) test.** CI=confidence interval. Groups with the same letter within the same climate region and management scenario are not significantly different at p=0.05.

| | | rainfed | | | | | irrigated | | | | | |
|---|---|---|---|---|---|---|---|---|---|---|---|---|
| Climate | Soil subset | 2.5% CI | Mean | 97.5% CI | Median | Group | 2.5% CI | Mean | 97.5% CI | Median | Group | Nutrient management |
| Tropic | $CV_{tot}$ | 40.612 | 41.155 | 41.705 | 40.518 | A | 36.332 | 36.649 | 36.970 | 36.386 | A | no nutrient input (no-nut) |
| | $CV_{dom}$ | 16.280 | 16.498 | 16.718 | 14.004 | B | 11.728 | 11.831 | 11.934 | 12.238 | C | |
| | $CV_{aw}$ | 16.399 | 16.618 | 16.840 | 14.053 | B | 12.069 | 12.175 | 12.281 | 12.221 | B | |
| Arid | $CV_{tot}$ | 76.210 | 77.996 | 79.824 | 78.220 | A | 41.295 | 41.696 | 42.101 | 41.861 | A | |
| | $CV_{dom}$ | 57.286 | 58.629 | 60.003 | 62.955 | B | 16.554 | 16.715 | 16.878 | 16.330 | B | |
| | $CV_{aw}$ | 55.436 | 56.736 | 58.065 | 61.045 | B | 15.912 | 16.067 | 16.223 | 15.692 | C | |
| Temperate | $CV_{tot}$ | 44.885 | 45.502 | 46.128 | 45.000 | A | 38.560 | 38.917 | 39.277 | 38.223 | A | |
| | $CV_{dom}$ | 21.986 | 22.288 | 22.595 | 20.290 | B | 14.183 | 14.314 | 14.447 | 14.765 | B | |
| | $CV_{aw}$ | 21.506 | 21.802 | 22.102 | 19.991 | B | 14.298 | 14.430 | 14.563 | 14.537 | B | |
| Cold | $CV_{tot}$ | 38.018 | 38.590 | 39.171 | 40.813 | A | 28.637 | 28.935 | 29.237 | 32.222 | A | |
| | $CV_{dom}$ | 23.711 | 24.068 | 24.430 | 22.620 | B | 14.279 | 14.427 | 14.578 | 14.531 | B | |
| | $CV_{aw}$ | 22.842 | 23.186 | 23.535 | 22.000 | C | 13.741 | 13.884 | 14.029 | 13.878 | C | |
| Tropic | $CV_{tot}$ | 29.113 | 29.537 | 29.967 | 28.663 | A | 23.301 | 23.548 | 23.797 | 23.540 | A | business-as-usual nutrient input (bau-nut) |
| | $CV_{dom}$ | 15.604 | 15.831 | 16.062 | 13.874 | B | 10.496 | 10.607 | 10.719 | 11.063 | B | |
| | $CV_{aw}$ | 15.404 | 15.628 | 15.856 | 13.557 | B | 10.597 | 10.709 | 10.823 | 10.967 | B | |
| Arid | $CV_{tot}$ | 67.682 | 69.379 | 71.118 | 70.642 | A | 17.685 | 17.965 | 18.249 | 16.997 | A | |
| | $CV_{dom}$ | 56.385 | 57.799 | 59.248 | 64.624 | B | 10.176 | 10.337 | 10.500 | 10.295 | B | |
| | $CV_{aw}$ | 53.867 | 55.217 | 56.602 | 60.872 | C | 9.864 | 10.020 | 10.179 | 9.856 | C | |
| Temperate | $CV_{tot}$ | 29.887 | 30.389 | 30.899 | 30.048 | A | 13.314 | 13.521 | 13.730 | 13.336 | A | |
| | $CV_{dom}$ | 19.812 | 20.144 | 20.482 | 19.734 | B | 8.370 | 8.499 | 8.631 | 8.693 | B | |
| | $CV_{aw}$ | 18.990 | 19.309 | 19.633 | 18.855 | C | 8.239 | 8.366 | 8.496 | 8.664 | B | |
| Cold | $CV_{tot}$ | 30.686 | 31.170 | 31.661 | 32.446 | A | 15.083 | 15.283 | 15.485 | 15.503 | A | |
| | $CV_{dom}$ | 23.628 | 24.001 | 24.379 | 23.503 | B | 11.897 | 12.054 | 12.214 | 11.965 | B | |
| | $CV_{aw}$ | 22.697 | 23.055 | 23.419 | 22.587 | C | 11.422 | 11.573 | 11.727 | 11.390 | C | |
| Tropic | $CV_{tot}$ | 12.225 | 12.488 | 12.757 | 11.442 | A | 4.014 | 4.045 | 4.076 | 3.975 | A | sufficient nutrient input (high-nut) |
| | $CV_{dom}$ | 9.837 | 10.048 | 10.265 | 7.653 | B | 4.002 | 4.033 | 4.064 | 3.942 | A | |
| | $CV_{aw}$ | 9.480 | 9.684 | 9.892 | 7.438 | C | 3.813 | 3.843 | 3.872 | 3.764 | B | |
| Arid | $CV_{tot}$ | 65.498 | 67.131 | 68.804 | 70.409 | A | 7.797 | 7.929 | 8.062 | 7.624 | A | |
| | $CV_{dom}$ | 61.109 | 62.633 | 64.194 | 67.671 | B | 7.515 | 7.642 | 7.771 | 7.456 | B | |
| | $CV_{aw}$ | 58.148 | 59.598 | 61.083 | 63.544 | C | 7.131 | 7.252 | 7.374 | 7.063 | C | |
| Temperate | $CV_{tot}$ | 24.800 | 25.271 | 25.750 | 26.971 | A | 5.377 | 5.444 | 5.512 | 5.002 | A | |
| | $CV_{dom}$ | 19.547 | 19.918 | 20.296 | 20.828 | B | 5.460 | 5.529 | 5.597 | 5.104 | A | |
| | $CV_{aw}$ | 18.521 | 18.873 | 19.231 | 19.700 | C | 5.191 | 5.255 | 5.321 | 4.831 | B | |
| Cold | $CV_{tot}$ | 29.772 | 30.243 | 30.722 | 31.239 | A | 10.611 | 10.763 | 10.917 | 10.335 | B | |
| | $CV_{dom}$ | 23.970 | 24.349 | 24.735 | 24.353 | B | 10.895 | 11.051 | 11.209 | 10.403 | A | |
| | $CV_{aw}$ | 23.018 | 23.382 | 23.752 | 23.516 | C | 10.372 | 10.521 | 10.672 | 9.912 | B | |

**Supplementary Table 3: Statistical coefficients for ANOVA, Tukey's HSD and Kendall's tau.** CI=confidence interval. Groups with the same letter within the same climate region and management scenario are not significantly different at p=0.05. The tau value and the associated p value indicate whether two samples are rank correlated, which is the case if tau≠0 and p<0.01.

| Climate | Nutrient management | ANOVA / Tukey's HSD | | | | | $CV_{tot}$ ratio bau-nut:no-nut | | Kendall's tau-Test | | | Water supply |
|---|---|---|---|---|---|---|---|---|---|---|---|---|
| | | 2.5% CI | Mean | 97.5% CI | Median | Group | Mean ratio | Median ratio | tau | p value | z value | |
| Tropic | bau-nut | 23.283 | 23.548 | 23.815 | 23.540 | A | 0.64 | 0.65 | -0.63 | <0.01 | -92.59 | irrigated |
| | no-nut | 36.238 | 36.649 | 37.065 | 36.386 | B | | | | | | |
| Arid | bau-nut | 17.962 | 18.230 | 18.502 | 17.174 | A | 0.43 | 0.41 | -0.59 | <0.01 | -67.53 | |
| | no-nut | 41.471 | 42.089 | 42.717 | 42.088 | B | | | | | | |
| Temperate | bau-nut | 13.310 | 13.521 | 13.735 | 13.336 | A | 0.35 | 0.35 | -0.62 | <0.01 | -77.40 | |
| | no-nut | 38.311 | 38.917 | 39.532 | 38.223 | B | | | | | | |
| Cold | bau-nut | 15.088 | 15.297 | 15.509 | 15.510 | A | 0.53 | 0.48 | -0.54 | <0.01 | -67.38 | |
| | no-nut | 28.562 | 28.957 | 29.358 | 32.223 | B | | | | | | |
| Tropic | bau-nut | 29.324 | 29.694 | 30.068 | 28.747 | A | 0.72 | 0.71 | -0.53 | <0.01 | -77.91 | rainfed |
| | no-nut | 40.994 | 41.510 | 42.034 | 40.734 | B | | | | | | |
| Arid | bau-nut | 87.031 | 88.579 | 90.154 | 95.163 | A | 0.91 | 0.95 | 0.03 | <0.01 | 3.74 | |
| | no-nut | 95.127 | 96.818 | 98.540 | 100.421 | B | | | | | | |
| Temperate | bau-nut | 30.750 | 31.168 | 31.591 | 30.552 | A | 0.67 | 0.67 | -0.16 | <0.01 | -20.10 | |
| | no-nut | 45.713 | 46.334 | 46.964 | 45.691 | B | | | | | | |
| Cold | bau-nut | 31.492 | 31.956 | 32.427 | 33.041 | A | 0.81 | 0.80 | -0.38 | <0.01 | -47.24 | |
| | no-nut | 38.834 | 39.407 | 39.987 | 41.406 | B | | | | | | |

**Supplementary Table 4: Percentiles of nitrogen and water application in scenario high-nut-irr corresponding to Supplementary Fig. 18.**

| Agricultural input | 25% | 50% | 75% | 90% |
|---|---|---|---|---|
| Total nitrogen appl. [kg $ha^{-1}$ $a^{-1}$] | 111.84 | 163.67 | 224.38 | 278.04 |
| Total irrigation vol. [mm $a^{-1}$] | 67.58 | 155.01 | 292.32 | 439.57 |

**Supplementary Discussion 1: Soil factors contributing to differences in climate change impacts**

Potential biomass accumulation is mainly a function of incoming solar radiation and heat unit accumulation (see equations (9) and (10) in paper). Hence, an increase in temperature results in faster maturing and allows for less time to accumulate biomass, which is one of the major temperature-related climate change effects on crop yields in the model (c.f. Folberth *et al.*[5]). The state of nutrient supply in contrast can increase or attenuate the model's sensitivity to climate-related stresses as on each day only the major stress related to nutrients, temperature, water or aeration is selected to limit the potential biomass increase. Water stress limits in addition yield formation during flowering, which is reflected in the water-stress adjusted harvest index ($HIA^*$) through which yield is derived from biomass at harvest (see equations (11) and (12) in paper). Together with atmospheric $CO_2$ fertilization, which allows for more rapid biomass accumulation, the complexity of these factors renders the exact evaluation of the soil's and climate's influences on plant growth across extended time periods within and across individual grid cells difficult.

We have further evaluated possible soil- and climate-related causes for yield changes in two contrasting grid cells to illustrate basic interactions between soil and climate affecting plant growth and yield formation. Both cases have been selected randomly as drivers for yield changes in a grid cell are *a priori* not known and can most often only be identified by analyzing daily simulation outputs. Still, both are related to soil hydrology. In the first example, the least suitable soil type renders maize yields on average more vulnerable to adverse climate change impacts, which is most often the case in major FPUs (Figure 5). The less frequent case that the presently most suitable soil type renders the crop more vulnerable to adverse climate change impacts is shown in the second example. Analogue to the main paper, simulations were driven by the climate datasets WFDEI.GPCC for the baseline period and HadGEM2-ES for a potential future climate change projection (see Methods).

We compare yields and environmental variables for a grid in FPU 13 (Chang Jiang West, CHN). Yields in FPU 13 show on average an increase on the most suitable soil type and a decrease on the least suitable one in the exemplary climate change impact assessment with management scenario bau-nut-rf (Figure 3 of the main article). The most suitable soil type in this grid cell is a *Haplic Chernozem* - considered one of the most fertile soils globally with substantial depth and OC content -, while the least suitable soil type is a *Mollic Leptosol* - a shallow stony soil.

During the future period, on the most suitable soil type yields increase on average by 11.4% and decrease by -10.8% on the least suitable one (not shown). Total biomass, however, increases by 7.6% and decreases by only -2.3% (Supplementary Fig. 4a). The larger difference in yields is caused by water deficits during yield formation as illustrated by the water-stress adjusted harvest index *HIA** (Supplementary Fig. 4b). While *HIA** is projected to be at least equally high and often substantially higher on the most suitable soil type in nearly all years of the future period as compared to the historic period, it is often substantially lower on the least suitable soil type.

During the 8$^{th}$ simulation year, biomass as well as $HIA^*$ are lower on the least suitable soil type in the future period causing a two-fold impact on crop yield. During the past period, the evolution of biomass is nearly equal on both soil types (Supplementary Fig. 5). In the future period, this is the case during the first 30% of the exponential growth phase. Subsequently, biomass accumulation starts to level off on the least suitable soil type. On the most suitable soil type, biomass increases beyond the magnitude of the baseline period despite the shortened time until maturity. This can be attributed to

the increased level of atm. $CO_2$, which reaches about 520 ppm (+50% compared to the baseline) on average in this period.

Both the limited biomass accumulation and the limited yield formation are caused by water stress on the least suitable soil type in the future time period (Supplementary Fig. 6). This stress often takes values close to 1 (corresponding to complete growth inhibition) during the second half of the growing period. Water stress also occurs on the most suitable soil type, but less frequently and with a lower intensity. The water deficit is caused by a decrease in precipitation during the growing period and especially flowering, conjoined by an increase in temperature (Supplementary Fig. 7), which results in higher potential ET and hence plant water requirement.

Supplementary Figure 8 indicates how $HIA^*$ is impacted by the water use ratio (*WUR*; see Methods). During the historic period WUR is constantly >0.5 on both soil types, the threshold for limiting $HIA^*$, while it is most of the time <0.5 in the second half of the future time period. This causes $HIA^*$ to be about 75% lower on the least suitable soil type, corresponding to a loss of 75% of flowers in addition to the overall reduced biomass.

We can hence conclude that the shallow, rocky soil renders the crop here more vulnerable to adverse climate change impacts due to its low water storage capacity, while a more fertile and porous soil can buffer adverse changes in precipitation, while allowing higher biomass accumulation due to atm. $CO_2$ fertilization.

The opposite case of lower vulnerability or even a positive impact of climate change on the presently least suitable soil type occurs rarely and drivers are far more complex to disentangle. As an example we compare yields and environmental variables for a grid in FPU 19 (Orange, South Africa) in which the least suitable soil type is a *Leptosol* as in the preceding example and the most suitable soil type is a *Phaeozem*, which is rich in OC and has a finer pore space than the stony soil.

Yields decrease on the presently most suitable soil type in the future period by -18.9% (from 4.2 to 3.4 t $ha^{-1}$ as averaged over a 10-year period), while they slightly increase on the least suitable soil type by 13.3% (2.14 to 2.43 t $ha^{-1}$). Although the loss on the most suitable soil type is more dramatic than the yield gain on the least suitable one, absolute yield on the first are on average still higher than on the latter. Annual absolute yields and their dynamics differ strongly on the most suitable soil type as compared to those on the least suitable one during the historic period, but become quite similar in the future time period (Supplementary Fig. 9). Yields on the least suitable soil type are not always higher in the future period as compared to the historic, but exhibit lower fluctuations that finally result in a net yield gain.

Apparently, the crop cannot profit as much from atm. $CO_2$ fertilization on the initially more suitable soil as on the least suitable one. One reason for this is the change in precipitation patterns (Supplementary Fig. 10) and soil water storage as can be seen in simulation year nine, in which the least suitable soil type allows for even a slightly higher yield than the most suitable one. Total growing season precipitation is in both periods at the lower limit of suitability for rainfed maize cultivation with about 400-500 mm on average. In the particular simulation year analyzed here, the amount decreases in the future time period by 10% and is more evenly distributed.

A more evenly distribution of precipitation is in general considered more favorable. However, the presently more suitable soil type has a higher wilting point water content due to a larger volume of fine pores, which causes here less water to be available in the future period for the plant early and late in the growing season (Supplementary Fig. 11). This results in less plant evaporation during those periods (Supplementary Fig. 12), which in turn causes water stress and a reduction in harvest index.

Investigating the particular soil type and climate configuration in this grid cell reveals that also a presently more suitable soil may become (relatively) less productive under climate projections. This is, however, a rare case and occurs mostly in regions with adverse growing conditions that cause yields on both the most and least suitable soil types to be in the lower range of otherwise attainable yields.

**References**


1. Elliott, J. *et al*. *The Global Gridded Crop Model Intercomparison: data and modeling protocols for Phase 1 (v1.0).* Geosci. Model Dev. **8**, 261-277 (2015)
2. Monfreda, C., Ramankutty, N. & Foley, J.A. Farming the planet. Part 2: Geographic distribution of crop areas, yields, physiological types, and net primary production in the year 2000. *Glob. Biogeochem. Cycles* **22**, GB1022 (2008).
3. Portmann, F.T., Siebert, S. & Döll, P. MIRCA2000 - global monthly irrigated and rain-fed crop areas around the year 2000: a new high-resolution dataset for agricultural and hydrological modeling. *Glob. Biogeochem. Cycles* **24**, GB 1011 (2010).
4. Peel, M.C., Finlayson, B.L. & McMahon, T.A. Updated world map of the Koeppen-Geiger climate classification. *Hydrol. Earth Syst. Sci.* **11**, 1633-1644 (2007).
5. Folberth, C. *et al.* Effects of ecological and conventional agricultural intensification practices on maize yields in sub-Saharan Africa under potential climate change. *Environ. Res. Lett.* **9**, 044004 (2014).
